# Changes in movement, habitat use, and response to human disturbance accompany parturition events in bighorn sheep (*Ovis canadensis*)

**DOI:** 10.1186/s40462-023-00404-2

**Published:** 2023-07-04

**Authors:** Aidan Brushett, Jesse Whittington, Bryan Macbeth, John M. Fryxell

**Affiliations:** 1 Parks Canada, Banff National Park Resource Conservation, PO Box 900, Banff, AB T1L 1K2 Canada; 2 Department of Integrative Biology, University of Guelph, Guelph, ON N1G 2W1 Canada

**Keywords:** Bighorn sheep, *Ovis canadensis*, Parturition, Lambing, Wildlife management, Conservation, Hidden Markov, Resource selection

## Abstract

Parturition and the early neonatal period are critical life history stages in ungulates with considerable implications for population growth and persistence. Understanding the changes in behaviour induced by ungulate parturition is important for supporting effective population management, but reliably identifying birth sites and dates presents a challenge for managers. Rocky Mountain bighorn sheep (*Ovis canadensis canadensis*) are one such highly valued and ecologically important species in montane and subalpine ecosystems of Western North America. In the face of changing patterns of anthropogenic land use, wildlife managers increasingly require site-specific knowledge of the movement and habitat selection characteristics of periparturient sheep to better inform land use planning initiatives and ensure adequate protections for lambing habitat. We used movement data from GPS collared parturient (n = 13) and non-parturient (n = 8) bighorn sheep in Banff National Park, Canada to (1) identify lambing events based on changes in key movement metrics, and (2) investigate how resource selection and responses to human use change during the periparturient period. We fit a hidden Markov model (HMM) to a multivariate characterization of sheep movement (step length, daily home range area, residence time) to predict realistic lambing dates for the animals in our study system. Leave-one-out cross validation of our model resulted in a 93% success rate for parturient females. Our model, which we parameterized using data from known parturient females, also predicted lambing events in 25% of known non-parturient ewes in a test dataset. Using a latent selection difference function and resource selection functions, we tested for postpartum changes in habitat use, as well as seasonal differences in habitat selection. Immediately following lambing, ewes preferentially selected high-elevation sites on solar aspects that were more rugged, closer to escape terrain, and further from roads. Within-home range habitat selection was similar between individuals in different reproductive states, but parturient ewes had stronger selection for low snow depth, sites closer to barren ground, and sites further from trails. We propose that movement-based approaches such as HMMs are a valuable tool for identifying critical parturition habitat in species with complex movement patterns and may have particular utility in study areas without access to extensive field observations or vaginal implant transmitters. Furthermore, our results suggest that managers should minimize human disturbance in lambing areas to avoid interfering with maternal behaviour and ensure access to a broad range of suitable habitat in the periparturient period.

**Supplementary Information:**

The online version contains supplementary material available at 10.1186/s40462-023-00404-2.

## Background

Birth and the neonatal period are critical life history stages in ungulates that directly impact population dynamics, demography, and the long-term sustainability of populations [1–3]. For example, changes in offspring survival can affect annual recruitment and may cause fluctuations in abundance that are relevant to ungulate conservation and management, especially in smaller populations [1, 3, 4]. Understanding how spatiotemporal variation in resource abundance and predation risk influence the location, timing, and habitat selection surrounding birth events can help researchers understand mechanisms of population change [3, 5–7]. However, for many species, reliably identifying reproductive events presents a major methodological challenge for wildlife managers.

Historically, parturition in free-ranging ungulates has been monitored through radio-telemetry observations, vaginal implant transmitters, or other field-intensive methods, all of which can present limitations due to cost, bias, or ethical implications [8–10]. There is considerable value in further developing methods to remotely identify reproductive events, in particular through the use of GPS collar data. Parturition and offspring rearing can invoke changes in movement, social relationships, and habitat use that provide indirect signals that a reproductive event has occurred [11]. For example, since low offspring mobility considerably constrains maternal movement, researchers have succeeded in indirectly identifying parturition events from fine-scale GPS-collar data by screening for sudden decreases or distributional changes in movement parameters [11–13]. A wide variety of analytical approaches to identifying birth events have been proposed, largely on a trial basis, with the greatest success in species demonstrating a single, prolonged decrease in postpartum movement [11–16]. Methods are less developed for species with sporadic patterns of movement, such as bighorn sheep (*Ovis canadensis*), where the use of single change point analyses or simple movement variables (e.g., step length) may be inadequate for reliably discerning parturition events.

The applications of remotely determining parturition extend well beyond increasing the efficiency of wildlife monitoring programs. Investigating the characteristics of identified birth events has great importance for wildlife managers seeking to understand ecological processes associated with offspring birth and care, such as movement and habitat selection [3, 5, 17]. For example, parturition timing has implications for recruitment and offspring born earlier in the reproductive season often experience greater survival [18]. The habitat features of parturition sites have additional influences. Maternal behaviour and site use during neonatal care can be strongly affected by both the high nutritional demands imposed by lactation and the need for offspring safety from predation [19, 20]. Strategies for predator avoidance can decrease foraging opportunities, so the periparturient period is commonly associated with selection of sites that optimize potential trade-offs between the nutritional needs of mothers and the safety of their young [20, 21]. The relative importance of predator avoidance and forage availability depend in part on offspring mobility and the position of a species along the ‘hider-follower continuum’ [11, 16, 21]. ‘Hider’ species such as elk (*Cervus canadensis*) and deer (*Cervidae* spp.) have immobile but well-concealed young for a prolonged period, and often prioritize access to high-quality forage—even in areas of high predator density [8, 16]. ‘Follower’ species such as caribou (*Rangifer tarandus*) and bighorn sheep have mobile, semi-independent offspring within hours or days of birth, and often select habitat with greater emphasis on predator avoidance over immediate access to forage surrounding birth sites [22, 23].

Rocky Mountain bighorn sheep (*Ovis canadensis canadensis*; hereafter ‘bighorn sheep’ or ‘sheep’), are a well-studied follower species of conservation concern, but data gaps surrounding reproductive events may prevent effective population management and protection of periparturient habitat. In many areas, bighorn sheep have experienced historical declines due to disease, hunting, and human activity [24–26]. Amongst other vital rates, lamb birth and survival carry importance for the recovery of declining or at-risk bighorn sheep populations and can be influenced by the timing and location of birth events [7]. During the lambing season, ewes have been observed maintaining distinct, isolated lambing areas with high ruggedness and comparatively low predator densities [27, 28]. Indeed, general bighorn sheep habitat is strongly hypothesized to confer safety from predators: within montane and subalpine environments, bighorn sheep of all age classes demonstrate preferences for areas of high slope, visibility, ruggedness, and proximity to escape terrain [29–31].

Reproductive behaviour in in bighorn sheep may also be influenced by anthropogenic activity. In many ungulates, including bighorn sheep, mothers often avoid human settlements during the periparturient period because offspring in disturbed areas can suffer reduced survival [9, 32, 33]. Human infrastructure and recreation may also act as stressors by inducing avoidance behaviours or excluding parturient sheep from otherwise-suitable lambing habitat [17, 26]. Although less emphasis has been placed on the relationships between human-caused disturbance and habitat selection and lamb survival in bighorn sheep, previous studies have suggested variable effects of activity on adult bighorn sheep in high human-use areas. For instance, roadside areas and restored industrial sites (e.g., open-pit mines) can benefit sheep by generating mineral sources or novel habitat [34, 35]. However, bighorn sheep also habituate poorly to disturbances from helicopters, vehicles, and recreational traffic [36, 37], and lamb survival rates have been documented to be lower around human developments [38].

The goal of our study was to identify the presence and timing of lambing events in a population of bighorn sheep in a high-recreation area of Banff National Park, Canada, using movement data, and to assess differences in habitat selection between ewes with and without lambs with an emphasis on human disturbance. Little knowledge exists about lambing preferences in this region and stronger understanding of the sites used during the periparturient period is vital to support land-use planning. As well, since inferences about lambing habitat across studies can be limited due to disparities between the environmental attributes of study areas, wildlife managers require site-specific data on lambing events [9, 39, 40]. Our study contained three objectives. First, we developed a hidden Markov model (HMM) to identify periods of stationary behaviour in female bighorn sheep, which we used to predict the timing and location of lambing events. Second, we tested the hypothesis that the needs of ewe-lamb pairs during the periparturient period would invoke changes in maternal habitat use and selection that were distinct from non-parturient ewes. Finally, we tested the hypothesis that parturient ewes would be more negatively affected by human infrastructure than non-parturient ewes. We expected that parturient ewes would exhibit stronger selection for habitat features commonly associated with reduced predation risk (e.g., proximity to escape terrain) and reduced human disturbance (e.g., proximity to trails and roads).

## Methods

### Study area

**Fig. 1:**
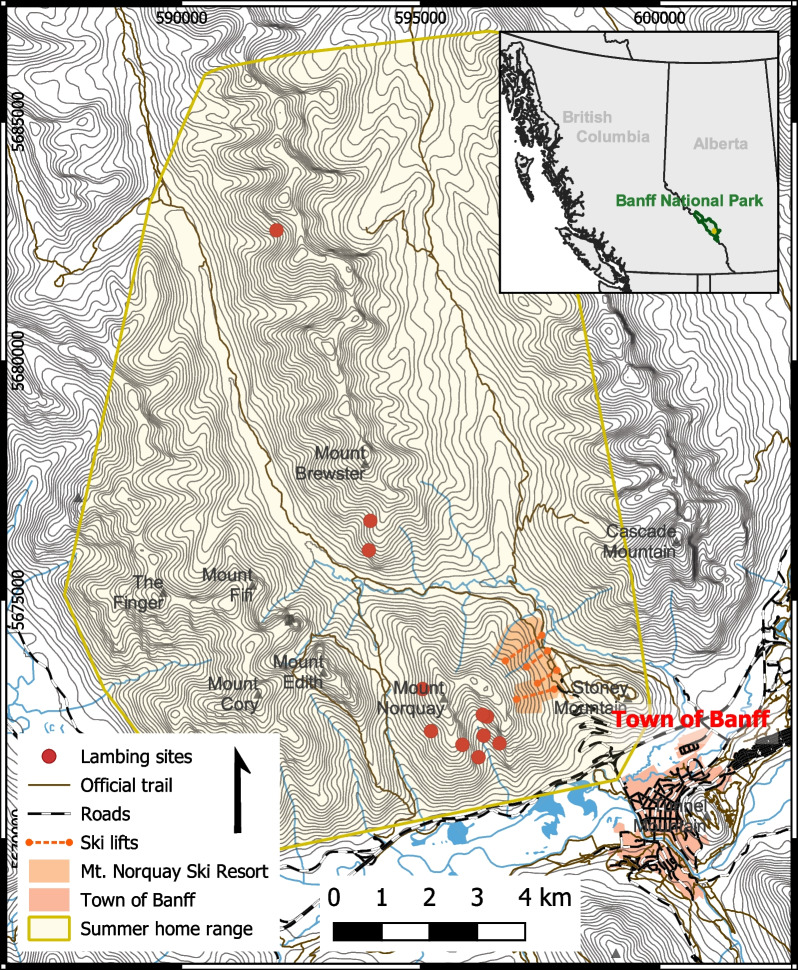
Summer home range of the 24 collared female bighorn sheep in our study and relevant human infrastructure. Home range was calculated from a 95% minimum convex polygon around all GPS locations from May 15–July 15. Red points indicate the locations corresponding to the lambing dates inferred for parturient ewes using a hidden Markov model

This study was conducted in Banff National Park, Alberta, a topographically diverse region along the eastern ranges of the Canadian Rocky Mountains. The bighorn sheep monitored in this study were located in a sub-region of Banff National Park centered on Mount Norquay (2455 m) and Mount Brewster (2859 m; Fig. 1). Elevation in the study area varied widely from valley bottoms to mountain peaks (approx. 1300–2859 m). Climate in Banff National Park was characterized by cold winters and brief, mild summers, with most precipitation occurring in spring. Vegetation was classified into montane, sub-alpine, and alpine ecoregions. The montane zone was dominated by forested areas of Engelmann spruce (*Picea engelmanii*), lodgepole pine (*Pinus contorta*), and aspen (*Populus tremuloides*), parklands, and grasslands. Subalpine and alpine zones—the primary habitat of Rocky Mountain bighorn sheep—were composed of subalpine fir (*Abies lasiocarpa*), subalpine larch (*Larix lyallii*), shrubby willows (*Salix* spp.), and grass and forb meadows. The primary predators of bighorn sheep in the area were wolves (*Canis lupus*) and cougars (*Puma concolor*), with additional occasional predation by grizzly bears (*Ursus arctos*) and black bears (*Ursus americanus*) [25]. Other ungulates in the park included mule deer (*Odocoileus hemionus*), white-tailed deer (*Odocoileus virginianus*), elk, moose (*Alces alces*), and mountain goats (*Oreamnos americanus*). Flora and fauna of the area are described in detail in Holroyd & Van Tighem [41].

The sheep in this study were year-round residents of Banff National Park and maintained distinct but spatially overlapping winter and summer ranges, with peak lambing occurring during approximately early- to mid-June [18]. All sheep included in this study used the same home range. The summer home range (May 15–July 15) covered an area of 159.1 km^2^ (Fig. 1). The grassy slopes of Mt. Norquay have high-value herbaceous communities that are recognized as an important convergence point for several bighorn sheep ranges during summer. The study area encompassed or neighboured a variety of human infrastructure, including the Town of Banff (pop. 8000), the Trans-Canada Highway, the Canadian Pacific Railway, and the Mount Norquay Ski Resort, a year-round recreational facility spanning the eastern slopes of Mt. Norquay. The study area experienced a wide variety of anthropogenic activity in the spring and summer that included sightseeing, hiking, scrambling, rock climbing, trail running, chairlift operations, helicopter flights, horse riding, and other activities (Fig. 1). All roads in the study area were paved, high-traffic roads. All trails were unpaved, with the primary users being hikers, trail runners, and rock climbers accessing alpine areas.

### Sheep capture and field observations

We captured a random sample of 16 female bighorn sheep in autumn 2019–2021 and fitted them with VHF-equipped GPS collars (VERTEX Lite IRIDUM; Vectronic Aerospace GmbH, Berlin, DE) with a two-hour fix rate. During the 2020, 2021, and 2022 lambing seasons (May 15–July 15), we performed regular (approximately weekly) ground observations on collared ewes to assess potential signs of pregnancy, recent birth, or lambs at heel. Several ewes were observed over multiple years, resulting in 24 ewe-years of reproductive data. Based on regular field observations, we classified each ewe-year as parturient (n = 13), non-parturient (n = 8), or uncertain (n = 3; see Additional file 1: T1 for all individual statuses). In confirmed parturient individuals, we estimated an approximate birth period based on the last observation pregnant, the first observation of lamb at heel, and visual estimates of lamb age, which we later used to assess the validity of the lambing dates inferred from our movement model.

### GPS error screening and characterization of movement

Positional errors from GPS collar data can strongly influence movement models and resource selection models and need to be removed before analysis [42, 43]. We identified erroneous collar fixes in our dataset using a series of rules adapted from Bjørneraas et al. [44]. First, we removed fixes classified as ‘2D’ (< 4 satellites used to determine location). Second, we removed fixes if their incoming travel speeds exceeded a reasonable maximum value, which we set at 5 km/h. Third, we removed fixes if their incoming and outgoing speeds exceeded 2 km/h (0.99 percentile of the dataset), and the cosine of the turning angle was less than −0.97. In all, we identified less than 0.25% of collar fixes as errors that were removed from the dataset. The total numbers of collar fixes used in subsequent analyses are presented in an additional file (see Additional file 1: T1).

To quantify maternal movement through the lambing season, we derived a multivariate characterization of movement that we hypothesized could signal lambing events. For every GPS collar position in our time series, we calculated three movement metrics: (1) the distance between successive collar fixes, or step length (*DIST*), (2) the residence time within a 100 m-radius (*RT100*), and (3) the day home range area (i.e., over a 24-h rolling window; *HR*). Residence time, adapted from Barraquand & Benhamou [45], was computed as the sum of the forward and backward time before an individual exited a 100 m radius of their given position for a period greater than two hours. Day home range area was determined from a 95% minimum convex polygon (MCP) around all collar fixes inside a 24-h window centered on an individual’s given position. We used a 24-h window to smooth over the effect of diurnal variations in movement on home range. Residence time and day home range were calculated using the *adehabitat* package in R v.4.1.1 [46].

### Hidden Markov model to identify lambing events

A hidden Markov model (HMM) is a form of statistical modelling that can be used to determine underlying latent behavioural states of animals based on positional, temporal, and environmental data associated with animal movements [47]. HMMs have been used to identify latent movement states in a wide variety of wildlife applications, but they have rarely been used to identify parturition events [48–51]. HMMs consider the probability of transitioning between states at the next timestep given the current state and associated covariate values [47]. To identify bighorn sheep lambing events, we first fit a HMM to known parturient ewes (May 15–July 15) using our three movement metrics (*DIST, RT100, HR)* as explanatory variables and specified a gamma error distribution using a log link for each variable. We specified three hidden states for the HMM putatively associated with three scales of sheep movement. We expected the first two hidden states to correspond to high movement (e.g., travelling & large-scale movements) and low movement (e.g., foraging & resting). We expected the third latent state to correspond to prolonged periods of non-movement or stationary behaviour—that is, a scale of movement distinctly lower than would be expected during normal rest or rumination. We expected this ‘non-movement’ state to be relatively unique to parturient ewes and act as a signal of birth events. We optimized our HMM and assigned a behavioural state to each timestep with the Viterbi algorithm using the *depmixS4* package in R [47]. We developed a decision rule based on our existing understanding of bighorn sheep biology to discern when a period of ‘non-movement’ exceeded a threshold that would signal a parturition event [25]. Specifically, we assigned parturition as the date at the beginning of the two-day period in which a ewe spent the greatest proportion of time in the ‘non-movement’ latent state, provided that greater than 50% of the movements were classified in the ‘non-movement’ state.

We evaluated the predictive value of our parameterized HMM separately for parturient and non-parturient individuals. First, we used Leave-One-Out Cross-Validation (LOOCV) to evaluate our HMM’s performance on parturient individuals. In each iteration of our cross-validation, we subset our dataset of 13 validated parturient ewes into a training set comprised of 12 individuals and a test set comprised of the remaining individual. We then fit our HMM using the movement data from the training set, applied the parameterized model to the remaining ewe and identified a lambing date—if there was one. We considered each iteration a success if (1) our model successfully predicted a lambing date, and (2) the lambing date aligned with the time period for birth determined from field observations. Furthermore, we applied our parameterized model from the full dataset (13 individuals) to our remaining data (i.e., non-parturient and unknown status) to assess our model’s out-of-sample predictive capabilities. In validated non-parturient individuals, we considered the model a success if no lambing date was identified.

### Comparison of prepartum and postpartum habitat use

Movement analyses and resource selection analyses are commonly used in tandem to contrast habitat selection across movement or behavioural states [52]. Accordingly, we delineated the data from parturient ewes into two putative behavioural states associated with the prepartum and postpartum period using the lambing dates inferred from our HMM. We then used a latent selection difference (LSD) function to test whether short-term habitat usage differed significantly between the prepartum and postpartum periods [20, 53]. LSD functions provide a measure of changes in habitat use. Since they do not characterize the null distribution of habitat availability, inferences cannot be extended to habitat selection when levels of availability change—such as when animals travel to a new, distinct area [53]. Locations used in the LSD functions were drawn from a 15-day period before and after the inferred lambing date, for a total of 30 days of data per individual. We estimated our LSD function by fitting a mixed-effect logistic regression of behavioural state (prepartum = 0, postpartum = 1) across eleven biologically relevant landscape covariates potentially associated with resource access, predator avoidance, and anthropogenic features (see Additional file 1: T2). Two covariates, distance to roads and distance to trails, were used to characterize to the most common forms of human use in the study area. To account for the diminishing influence of human disturbance with increasing distance, we transformed these variables using an exponential decay term such that locations greater than 500 m from a road or trail had little change in value (see Additional file 1: T2). To account for the non-independence of observations within an individual, we included a random intercept for each animal in the model [54].

### Comparison of habitat selection between parturient and non-parturient ewes

Habitat selection occurs on hierarchical scales [55, 56], and it is possible that parturient ewes exhibited habitat preferences within their summer range distinct from non-parturient ewes throughout the entirety of the lambing season, in addition to short-term effects in the immediate periparturient period. We tested for seasonal differences in habitat selection on the within-home range scale by fitting resource selection functions (RSF) separately for both reproductive statuses; individuals with uncertain reproductive status were excluded from the analysis.

RSFs estimate habitat selection by comparing the habitat characteristics of used sites and available sites [55, 57, 58]. We estimated within home range habitat selection by comparing habitat attributes of used vs. available sites within sheep home ranges. Since the same habitat was available to all ewes, we determined home range using a 95% MCP around all sheep locations during the lambing season. We took a random sample of available sites at a 10:1 ratio to used sites. To estimate our RSFs, we fit mixed-effect logistic regressions of site (available = 0, used = 1) to the same eleven landscape covariates as our LSD function (see Additional file 1: T2). To account for the non-independence of observations within an individual, we included a random intercept for each animal in the model [54]. To determine whether there were differences in habitat selection between reproductive statuses, we compared the confidence intervals for selection coefficients for each landscape covariate between parturient sheep and non-parturient sheep.

## Results

### Inference of lambing events from HMMs

**Fig. 2:**
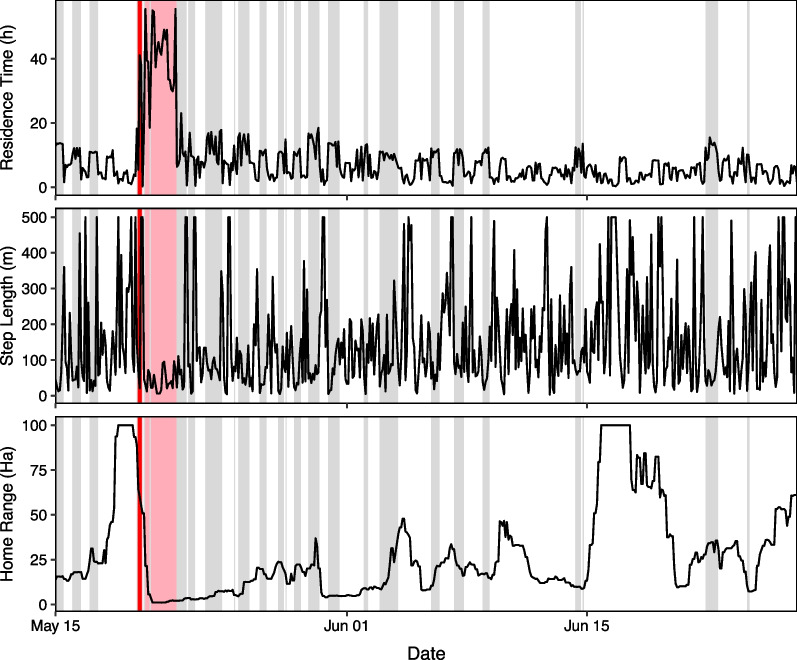
Estimated a posteriori hidden states (using the Viterbi algorithm) of B05-2020 based on a hidden Markov model fit to three response variables: step length, residence time, and day home range. The red hidden state corresponds to the ‘non-movement’ latent state associated with parturition, the grey state corresponds to the ‘low-movement’ latent state, and the white state corresponds to the ‘high-movement’ latent state. Day home range has been truncated at 100 Ha to improve scale. Using our decision rule, we identified a lambing date of May 20, 2020 (red line) which matched the lambing date estimated from field observations

**Fig. 3:**
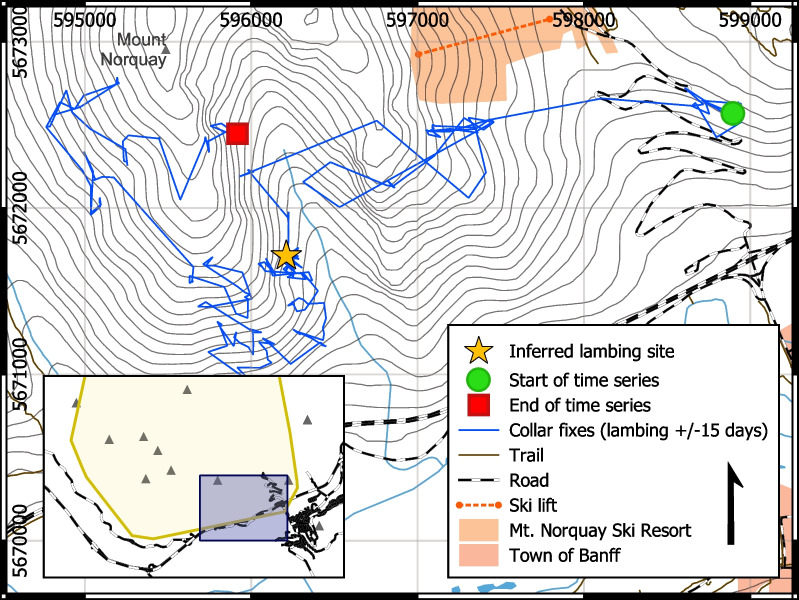
GPS-collar locations of ewe B05-2020 fifteen days before and after the lambing date inferred by a hidden Markov model (May 20, 2022), beginning and ending at the green circle and red square, respectively. The lambing date corresponded to a two-day (approx.) cluster of collar fixes within a small radius

The HMM classified lambing season movements of parturient into three hidden states associated with three rates of movement, which matched our expectation of movement attributes associated with bighorn sheep travel, rest/forage, and parturition. High values of residence time and low values of day home range area mapped closely to the ‘non-movement’ state. We provide full state probabilities and transition probabilities for our parameterized HMM in an additional file (see Additional file 1: T3). We did not observe notable effects of diurnal cycles in animal movement or rumination on the ‘non-movement’ latent state classifications. Using our decision rule, we successfully identified a lambing date for each parturient ewe (see Additional file 1: T1). Each lambing date preceded a brief but distinct period in which the ewe spent most of her time in the ‘non-movement’ latent state (Fig. 2, all figures in Additional file 2). High movement periods commonly preceded ‘non-movement’ states associated with parturition (Fig. 2). Spatially, each lambing date was associated with the entry point into a distinct cluster of collar fixes (Fig. 3). All thirteen inferred lambing dates fell within the range of dates in which lambing was known to occur, and matched estimated lamb age determined from field observations.

**Fig. 4:**
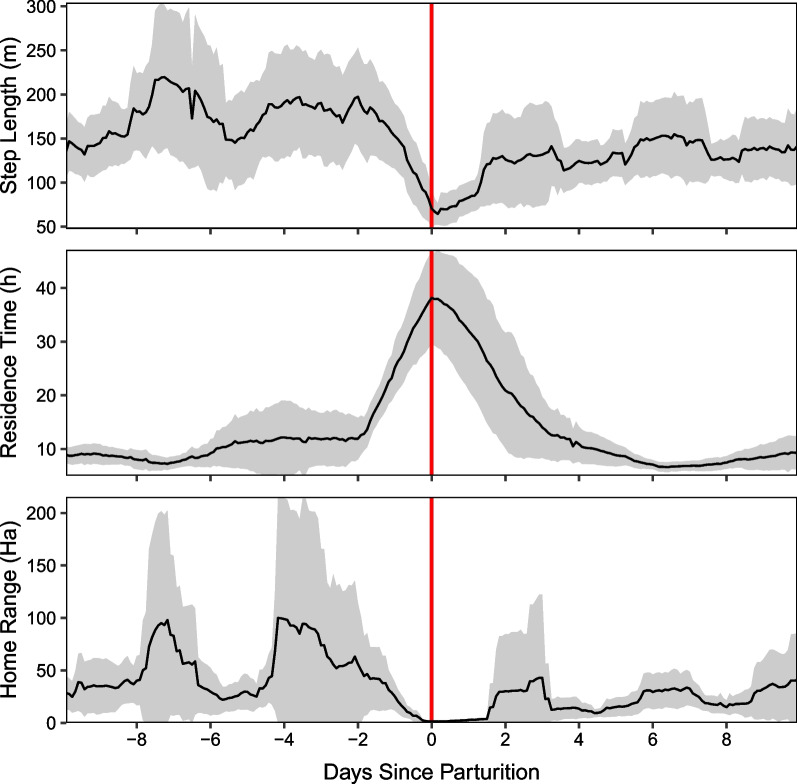
Aggregate movement trends for parturient bighorn sheep, aligned by inferred lambing date. Displayed values are the mean value of daily average movement metrics. Grey envelopes represent 95% confidence intervals. Generally, lambing events were preceded by an increase in home range and followed by a large reduction in movement rate for a period less than two days long

Overall, immediately following the lambing date, ewes demonstrated a sharp increase in daily average residence time to a maximum of 37.9 ± 8.8 h, a decrease in daily average step length to a minimum of 62.2 ± 10.5 m, and a decrease in day home range area to 1.9 ± 0.6 Ha (mean ± 2SE; Fig. 4). Lambing events were preceded by an increase in home range, although the magnitude and timing varied considerably among individuals. Spatially, ten of thirteen lambing sites were located on the southwest facing slopes of Mt. Norquay, at the bottom of the population’s home range (Fig. 1). Numerous individuals travelled large distances (up to 16 km) from nearby peaks (Mount Brewster, Cascade Mountain) to this area for lambing.

Each of the thirteen iterations used in the LOOCV successfully identified a lambing date for its respective test animal. Twelve of the predicted lambing dates agreed with those identified from the full dataset within ± 6 h and continued to align strongly with field observations. However, the predicted lambing date for one individual occurred 23 days earlier than expected. This lambing date was associated with another period of ‘non-movement’ and did not align with validated field observations, thus we considered this prediction incorrect. In total, we considered our approach to be successful for 92% (12/13) of parturient ewes. We provide lambing dates estimated in the LOOCV in an additional file (See Additional file 1: T1).

**Fig. 5:**
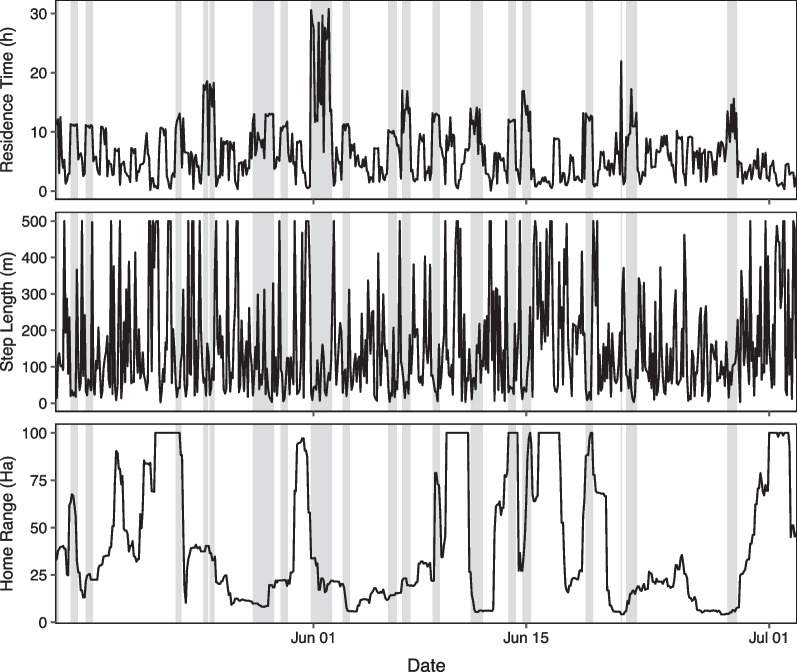
Movement metrics and estimated a posteriori hidden states (using the Viterbi algorithm) of B08-2022 (non-parturient) based on our trained hidden Markov model. The grey state corresponds to the ‘low-movement’ latent state, and the white state corresponds to the ‘high-movement’ latent state. No time was spent in the “non-movement” behavioural state associated with parturition. Day home range has been truncated at 100 Ha to improve scale. We did not identify a lambing date, which matched expectations from field observations

Our parameterized HMM also performed well for non-parturient ewes. The model did not identify lambing dates in 75% (6/8) of non-parturient ewes. Of the six sheep without identified parturition dates, five spent none of their time in the ‘non-movement’ latent state (Fig. 5) and one spent no longer than 4 h in the ‘non-movement’ state (all figures provided in Additional file 2). The HMM identified lambing dates for 67% (2/3) of ewes with uncertain reproductive status (see Additional file 1: T1). Both ewes appeared to be pregnant from field observations early in the lambing season, but we never confirmed offspring presence.

### Changes in habitat use and habitat selection

**Fig. 6:**
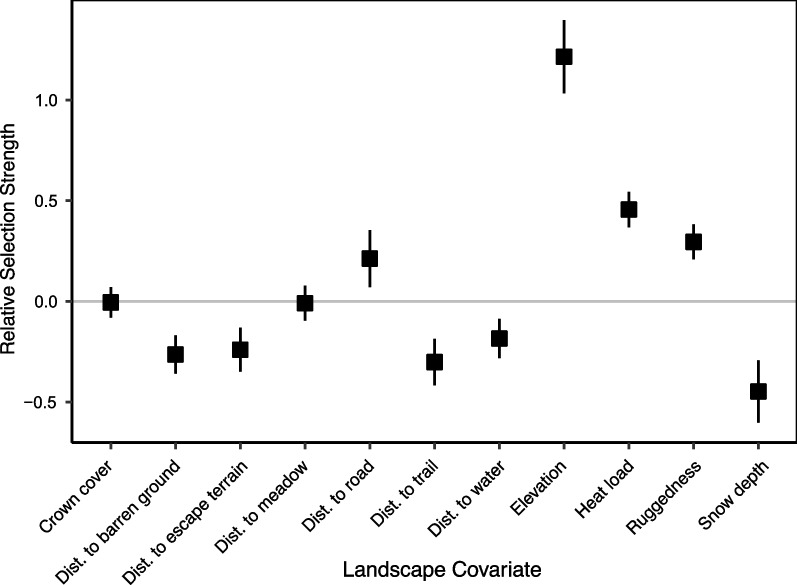
Coefficient estimates for each landscape covariate in a mixed-effect latent selection difference function modelling habitat use 15 days before and after the lambing dates inferred for parturient bighorn sheep ewes (n = 13). A random intercept was included in the model for each animal. Error bars are given as 95% confidence intervals. Positive values of relative usage for a “Distance to…” covariate imply selection for sites with a greater distance (i.e., further) from that landscape feature

Based on our LSD function, habitat use changed significantly following lambing for several landscape covariates. In the postpartum period, ewes used high-elevation, high-ruggedness sites that were closer to escape terrain and barren ground, moderately further from roads, and slightly closer to trails. They also used areas with higher ruggedness, higher heat load and lower snow depth (Fig. 6). The relative strength of the changes in habitat use was high (0.99) for elevation, but only moderate or weak (< 0.5) for all other landscape covariates (see Additional file 1: T4 for full model coefficients).

**Fig. 7:**
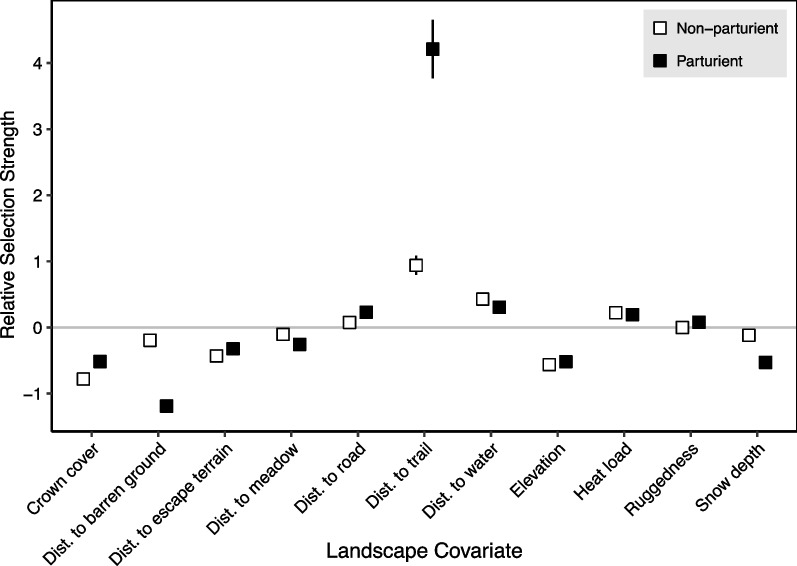
Selection coefficient estimates for each landscape covariate in use-availability resource selection functions modelling seasonal habitat selection (May 15–July 15) in parturient and non-parturient bighorn sheep ewes. A separate RSF was fit for each reproductive status. A random intercept was included for each animal in the models. Error bars are given as 95% confidence intervals. Positive values of relative usage for a “Distance to…” covariate imply selection for sites with a greater distance (i.e., further) from that landscape feature

Our RSFs revealed several overall patterns of habitat selection on the home range scale (Fig. 7). Within their summer home range, sheep demonstrated moderate selection for sites that were closer to barren ground, escape terrain, and herbaceous ground cover. They also selected habitat with lower elevation, crown cover, and snow depth. Sheep weakly selected areas further from roads, and strongly selected areas further from trails (Fig. 7). The direction of selection coefficients was the same for all landscape features. The effect size of selection coefficients was similar between reproductive statuses for most landscape features: the difference in relative selection strength was less than 0.25 for 8 of 11 covariates. However, parturient ewes showed much stronger selection than non-parturient ewes for sites closer to barren ground (*β*_part_ = –1.19, *β*_non-part_ = –0.19), much stronger selection for sites further from roads (*β*_part_ = *4.48*, *β*_non-part_ = *0.94*), and moderately stronger selection for low snow depth (*β*_part_ = –0.53, *β*_non-part_ = –0.12; see Additional file 1: T5 for full model coefficients).

## Discussion

Consistent with our predictions, parturition by bighorn sheep in our study induced a variety of important, identifiable changes in both movement parameters and habitat selection that have implications for bighorn sheep management and ecology. Our HMM used changes in movement to successfully identify lambing dates for parturient ewes that were strongly supported by field observations, previous studies, and qualitative knowledge of bighorn sheep behaviour in our study area. A latent selection difference functions provided evidence that observed postpartum changes in movement were accompanied by changes in the characteristics of used habitat. Our resource selection functions suggested that while most habitat preferences were the same among all ewes, there were also several distinct patterns in habitat selection at the within-home range scale that varied with reproductive status.

Parturient ewes reached their lowest movement rate soon after lambing, which is consistent with findings from earlier studies showing a marked decline in movement rates near parturition [12, 14–16, 59]. The rapid return of maternal movement to prepartum levels (Fig. 4) demonstrated that bighorn sheep exhibited only a brief decline in mobility following lambing. Notably, day home range remained low for an extended period (approximately 2 days or greater) following birth, suggesting a propensity for ewes to isolate in small home ranges during the immediate postpartum period, perhaps to facilitate mother-young bonding or reduce exposure to predators [60]. These results are consistent with the expected behaviour of a ‘follower’ ungulate species [21, 25].

In our test dataset, our approach predicted lambing dates in two of three ewes with uncertain reproductive status, both of which were strongly suspected to be pregnant prior to the lambing season. We also identified lambing dates in two of eight known non-parturient ewes, suggesting our model may be too inclusive in its identification of parturition periods. We propose three possible ecological explanations for these putative false positives. First, it is possible that a lamb was never observed during field observations due to early, undetected neonate mortality, such that ewes still underwent changes in movement associated with lambing. Second, the gregarious social structure of bighorn sheep may result in similar movement characteristics across female sheep of all reproductive statuses. Ewes, young-of-year, and yearlings often form nursery groups, so non-parturient ewes may follow parturient ewes to lambing sites, in turn demonstrating false signals of lambing [25]. Anecdotally, ewe-yearling pairs in our study area followed parturient ewes to lambing areas on numerous occasions. Third, the low-movement state identified in the HMM may occasionally signal other uncommon behaviours in nonparturient bighorn sheep, such as prolonged rest or particularly profitable bouts of foraging. Metrics such as residence time can identify any area of intensive use [45]. These reasons may also account for the non-movement period that resulted in a false negative result for one parturient ewe in our LOOCV. may also explain Additional fine-scale habitat and activity (accelerometry) data could improve the distinction between putative behavioural states and improve the predictive capabilities of our HMM in future [11].

HMMs have been used previously to identify phase-dependent changes in animal movement hypothesized to arise from changes in general behaviours, such as foraging, resting, and travelling [48, 49]. We applied a more specialized HMM to identify singular events of particular importance (lambing) based on episodic changes in movement with reasonable success. Parturient sheep in our study demonstrated a complex movement pattern and occasionally entered a ‘non-movement’ state multiple times in a single season. Our analytical approach provided a more flexible approach to discerning parturition events than single change point analyses based on conventional movement metrics such as step length or turning angle [12–14]. Asher et al. [50] also successfully adapted HMMs to identify calving dates in red deer (*Cervus elaphus*) hinds without the use of vaginal implant transmitters or prior knowledge of calving dates, similar to the constraints imposed on our study. While we were unable to directly compare inferred and precise true lambing dates, our results nonetheless suggest that HMMs are an effective tool for remotely identifying lambing dates in bighorn sheep. Recently, supervised machine learning approaches have been developed with similar predictive capabilities, but they have generally been applied to species with prolonged decreases in movement, or in study systems with a high amount of validated training and test data [11, 16, 61]. Our HMM approach may have particular utility for wildlife managers that are limited by small sample sizes or broad validation data. However, given our rate of false positives, the complex social structure of bighorn sheep, and the very short-term changes in movement during lambing, we suggest that some level of prior knowledge of reproductive status may still be required to reliably predict true lambing events. To potentially improve the ability of our approach to discriminate between parturient and nonparturient individuals, future studies may also wish to increase the control over the HMM-optimizing process by establishing a priori state probabilities or transition probabilities for a ‘parturition’ latent state using movement data from known lambing periods. Similarly, another avenue of research would be to identify parturition events with models that summarize movement attributes over longer time periods and more importantly prohibit multiple transitions to parturient movement states through constraints on transition probabilities. If successful, such models could more reliably identify pre-parturient, parturient, and post-parturient classes of movement and better distinguish parturient from non-parturient ewes.

The results from the second component of our study suggest that in addition to changes in movement, several changes in habitat use and selection occur during the lambing season that are important to bighorn sheep reproduction. Most of these changes occurred on short time scales and reflected a propensity to isolate with vulnerable young in small areas. During the two-week postpartum period, ewes used high-elevation sites on solar aspects that were moderately more rugged, and closer to escape terrain. These findings align with numerous previous studies that have suggested ewes seek isolation in extreme habitat during lambing and the early neonatal period [25]. The characteristics of postpartum habitat likely provide mother–offspring pairs with additional attempted opportunities for avoidance of predators such as wolves and grizzly bears, even at the expense of forage quality. Increased use of solar aspects and areas of low snow cover may also help minimize the potential losses of forage productivity and provide a thermal environment conducive to lamb growth and survival [30]. Indeed, these short-term changes in habitat use may facilitate survival in neonatal sheep, which can be as high as 97% during the first two weeks following birth [18, 62]. Several of these changes in habitat attributes—namely, selection for low snow depth and areas closer to rocky ground—were also apparent on the within-home range scale, suggesting that foraging opportunities and rocky or high-visibility sites may continue to carry importance throughout the lambing season.

However, we found that aside from these differences, seasonal habitat selection was mostly consistent for parturient and non-parturient ewes. On the within-home range scale, ewes exhibited strong patterns of habitat selection that aligned with known preferences of bighorn sheep [29–31]. Conventional bighorn sheep habitat, which is already highly specialized, is likely adequate to meet most requirements of mothers and lambs. Nursery groups form fluidly for much of the summer season [25] and parturient ewes in our study were observed joining nursery groups as early as one week following lambing. Thus, it is likely that parturient and non-parturient sheep often travel together and access similar spaces, supporting the conclusion that ewes of all reproductive statuses use largely similar habitats throughout the summer season.

Perhaps our most notable finding was that anthropogenic landscape characteristics appeared to have a stronger, net negative affect on parturient sheep, which used areas further away from roads immediately following lambing and selected against proximity to trails and roads in our RSFs. Curiously, our latent selection difference analysis suggested that sheep used areas that were closer to trails following lambing. We note that the RSF analysis, which accounted for habitat availability, found strong avoidance of trails. These results suggest that lambing sites were slightly closer to trails during lambing, but sheep continued to avoid areas near trails overall. Additionally, many identified lambing and postpartum sites in our study occurred in isolated alpine terrain features, where even nearby trails were geographically separated by impassable alpine ridges. Selection against roads was weaker than selection against trails, which may have been for several reasons. For example, a known mineral lick on the Mount Norquay Road may have contributed to this decrease in avoidance behaviour. Mineral licks exert a strong draw over bighorn sheep of all ages and reproductive classes during the spring and summer, even at the expense of increased predation risk or human disturbance [27, 28]. Furthermore, an impermeable wildlife fence bordering the Trans-Canada Highway may have mediated negative effects of the highway on space use. Additionally, wolves in our study area frequently used trails to increase travel efficiency [49], so it is possible that strong negative selection for trails by parturient bighorn sheep was in part due to predator presence on these linear features, in addition to high human use. Nonetheless, our findings suggest that human disturbance exerted a stronger, net-negative influence on parturient sheep.

Given the overall similarity of selection for natural landscape attributes for parturient and non-parturient females, human-avoidant behaviours may exclude mother-young pairs from high quality habitat at the within-home range scale. Indeed, a long-term study by Wiedmann & Bleich [26] found that the expansion of recreational infrastructure into bighorn sheep habitat led to lower recruitment rates, reduced lambing site fidelity, and eventual abandonment of former lambing habitat by ewes altogether. Purported positive benefits of human infrastructure discussed in other works [9, 34, 63] were not realized in our study area. Our findings add to a larger body of literature which has documented variable changes in habitat selection among populations and species [20, 21, 64–66]. We were limited in that our habitat data does not establish a causal relationship between habitat selection and forage quality, predator avoidance, or human avoidance. Incorporating more sophisticated landscape covariates into resource selection models (see Viejou et al. [20] for examples) may help establish a clearer understanding of the ecological influences on lambing behaviour and fitness.

Ten of thirteen inferred lambing sites occurred in a common area at the southern extent of the summer range. One ewe in our study lambed twice, in nearly identical locations each year. Bighorn sheep often demonstrate high lambing site fidelity within their home range, which further highlights the importance of protecting known lambing areas [26].

## Conclusions

We demonstrated that HMMs can be a valuable tool for determining the timing and location of parturition events. They may have particular relevance in species with complex movement patterns and study systems lacking intensive in-field observations for every individual, due to their ability to incorporate multivariate movement parameters and varying levels of a priori knowledge, respectively. Using movement data as a screening tool may also drastically improve the efficiency with which managers acquire validated field data about lambing events, in turn creating opportunities for more sophisticated population-level analyses. Using this approach, we determined that Rocky Mountain bighorn sheep undergo distinct changes in movement and habitat use during lambing that carry implications for wildlife management. Despite moderate levels of human use throughout most of our study area compared to other regions, parturient sheep were negatively affected by the presence of trails and roads. The effect of human disturbance on lambing may be mitigated through careful land-use planning that minimizes the encroachment of recreational development into existing bighorn sheep habitat.

## Supplementary Information

**Additional file 1.** Sheep metadata and model parameters. This file contains additional data about the reproductive status of each sheep, mortalities/collar removals that affect the continuity of data, and the number of collar fixes used in each analysis. Also included are the parameters from our optimized HMM, LSD function, and RSFs and a description of each landscape covariate used in habitat selection analyses.

**Additional file 2.** HMM results for each individual sheep. This file contains the figures with the movement metrics, predicted latent states, and lambing datefor each individual sheep in the test and training datasets, organized by reproductive status.

## Data Availability

The datasets and scripts supporting the findings of this article are available in the Git repository: https://github.com/aidan-brushett/Brushett-et-al-2023_bighorn-sheep-lambing.

## Abbreviations

HMM: Hidden Markov model
LOOCV: Leave-one-out cross-validation
LSD: Latent selection difference
RSF: Resource selection function
MCP: Minimum convex polygon
