# Supplementary material for: Changes in movement, habitat use, and response to human disturbance accompany parturition events in bighorn sheep (Ovis canadensis)
Source: Mov Ecol. 2023 Jul 4;11:36. doi: 10.1186/s40462-023-00404-2 (PMC10318713; doi:10.1186/s40462-023-00404-2)
Supplement: Supplementary file 1 — Additional file 1. Sheep metadata and model parameters. This file contains additional data about the reproductive status of each sheep, mortalities/collar removals that affect the continuity of data, and the number of collar fixes used in each analysis. Also included are the parameters from our optimized HMM, LSD function, and RSFs and a description of each landscape covariate used in habitat selection analyses. [file 40462_2023_404_MOESM1_ESM.pdf]

*Table 1. Sample sizes and lambing dates used in movement and habitat analyses*

| Sheep ID | Inferred Lambing | LOOCV Lambing | Number of Fixes | | Notes |
|---|---|---|---|---|---|
| | | | HMM, RSF | LSD | |
| **Parturient** | | | | | |
| B04 | 2020-05-26 | 2020-05-26 | 737 | 357 | |
| B05 | 2020-05-20 | 2020-05-20 | 742 | 360 | |
| B05 | 2022-05-20 | 2022-05-19 | 531 | 358 | Killed on June 28, 2022 by cougar |
| B08 | 2020-06-04 | 2020-06-04 | 744 | 361 | |
| B09 | 2020-06-03 | 2020-06-03 | 742 | 359 | |
| B16 | 2021-06-13 | 2021-06-13 | 741 | 357 | |
| B16 | 2022-05-31 | 2022-05-31 | 741 | 359 | |
| B17 | 2022-05-22 | 2022-05-22 | 742 | 357 | |
| B19 | 2021-06-11 | 2021-06-11 | 741 | 358 | |
| B22 | 2021-06-04 | 2021-06-04 | 740 | 359 | |
| B26 | 2021-07-02 | 2021-07-02 | 739 | 357 | |
| B51 | 2022-05-30 | 2022-05-31 | 744 | 360 | |
| B60 | 2022-06-05 | 2022-06-28 | 744 | 361 | |
| **Uncertain** | | | | | |
| B08 | *2021-05-24* | - | 738 | - | |
| B18 | *2021-05-24* | - | 740 | - | |
| B18 | *2022-05-16* | - | 744 | - | |
| **Non-parturient** | | | | | |
| B02 | - | - | 743 | - | |
| B05 | - | - | 742 | - | |
| B08 | - | - | 607 | - | Collar removed on July 4, 2022 |
| B17 | *2021-07-04* | - | 730 | - | |
| B19 | - | - | 354 | - | Killed on June 13, 2022 by cougar |
| B22 | - | - | 739 | - | |
| B23 | *2022-05-19* | - | 294 | - | Collar removed on June 8, 2022 |
| B61 | - | - | 734 | - | |

*Number of collar fixes used in a hidden Markov model and resource selection analyses for parturient ewes, non-parturient ewes, and ewes of unknown reproductive status. The number of fixes varies slightly by individual due to erroneous fixes, collar removal by wildlife managers, or predation events. Italicized lambing dates are either false positive results (i.e., in known non-parturient animals) or unvalidated (i.e., in animals with unknown reproductive status).*

***Table 2. Landscape covariates used in habitat selection analyses***

| Variable | Abbreviation | Description |
|---|---|---|
| Heat load | *heatload* | Relative measure (-1 to 1) of solar radiation, based on transformation of aspect (maximum at SSW aspect) and slope. |
| Distance to escape terrain | *descp* | Distance to escape terrain in meters, defined as areas with slope >70% and patch size >0.7 Ha (Poole et al., 2016). |
| Distance to barren ground | *dbarren* | Distance to barren ground (i.e., exposed terrain) in meters, derived from BC Vegetation Resource Inventory (Sandvoss et al., 2005) |
| Distance to meadow | *dmeadow* | Distance to patches of herbaceous ground cover in meters, derived from BC Vegetation Resource Inventory (Sandvoss et al., 2005) |
| Distance to road | *droad* | Distance to primary, secondary and tertiary roads in meters. Transformed using an exponential decay function $droad=1-e^{-0.01x}$, where $x$ was the distance to roads in meters. |
| Distance to trail | *dtrail* | Distance to official Banff National Park recreational trails, transformed using an exponential decay function: $droad=1-{}^{e-0.01x}$, where $x$ was the distance to trails in meters. |
| Distance to water | *dwater* | Distance to perennial water sources, including streams, ponds, and lakes in meters. Derived from Alberta Environment and Parks Base Hydrography Single Line Network (Government of Alberta, 1996) and BC Vegetation Resource Inventory (Sandvoss et al., 2005) |
| Elevation | *elevation* | Elevation, derived from 30x30 m DEM in QGIS 3.16. |
| Crown cover | *crowncover* | Percent canopy cover, remotely sensed (McDermid et al., 2009) |
| Snow depth | *snowdepth* | Mean annual snow depth derived from SnowCast Global Environmental Multiscale (GEM) model (Vionnet et al., 2021). |
| Ruggedness | *vrm* | Index of terrain ruggedness based on orthogonal vector dispersion, derived from 30x30 m DEM in QGIS 3.16 (Sappington et al., 2007). |

*Landscape covariates and data sources used for latent selection difference functions, resource selection functions, and step selection function models to detect changes in habitat use and selection of parturient and non-parturient bighorn sheep. All covariates are measured at a 30 m spatial resolution.*

***Table 3. Hidden Markov model parameters***

**[A] Emission Parameters**

| | ***DIST*** | ***RT100*** | ***HR*** |
|---|---|---|---|
| | Intercept | Intercept | Intercept |
| State 1 | -2.119 | 1.436 | -0.163 |
| State 2 | -3.259 | 2.495 | -1.326 |
| State 3* | -3.432 | 3.714 | -2.647 |

**[B] Transition Probabilities**

| | to State 1 | to State 2 | to State 3* |
|---|---|---|---|
| State 1 | 0.905 | 0.086 | 0.009 |
| State 2 | 0.203 | 0.764 | 0.033 |
| State 3* | 0.068 | 0.15 | 0.781 |

*[A] Response variable parameters and [B] transition matrix parameters from a hidden Markov model parameterized using the movement data of parturient ewes (n=13) from May 15–July 15. Step length (DIST) and residence time (RT100) were fit with a gamma error distribution with a log-link function. We assumed the shape and scale of the gamma distributions were the same among all three hidden states. Home range (HR) was fit with a Gaussian distribution. State 2 (*) in our HMM corresponded to the latent low-movement behavioural state that we used to infer lambing dates.*

***Table 4. Latent selection difference function model parameters***

| **Covariate** | **β** | **SE** | **p** |
|---|---|---|---|
| *(intercept)* | -0.016 | 0.160 | 0.922 |
| *heatload* | 0.472 | 0.045 | <0.001 |
| *descp* | -0.225 | 0.056 | <0.001 |
| *dbarren* | -0.281 | 0.050 | <0.001 |
| *dmeadow* | 0.011 | 0.044 | 0.799 |
| *droad* | 0.223 | 0.072 | 0.002 |
| *dtrail* | -0.097 | 0.040 | 0.015 |
| *dwater* | -0.079 | 0.046 | 0.081 |
| *elevation* | 0.987 | 0.080 | <0.001 |
| *crowncover* | -0.003 | 0.039 | 0.929 |
| *snowdepth* | -0.387 | 0.078 | <0.001 |
| *vrm* | 0.301 | 0.044 | <0.001 |

*Coefficient estimates for each landscape covariate in a mixed-effect latent selection difference function modelling habitat use 15 days before and after the lambing dates inferred for parturient bighorn sheep ewes (n=13). A random intercept was included in the model for each animal.*

***Table 5. Resource selection function model parameters***

| Covariate | β | | SE | | p | |
|---|---|---|---|---|---|---|
| | **Part.** | **Non-Part.** | **Part.** | **Non-Part.** | **Part.** | **Non-Part.** |
| *(intercept)* | *-3.864* | *-3.472* | *0.040* | *0.131* | *<0.001* | *<0.001* |
| heatload | 0.193 | 0.223 | 0.013 | 0.017 | <0.001 | <0.001 |
| descp | -0.321 | -0.430 | 0.030 | 0.036 | <0.001 | <0.001 |
| dbarren | -1.190 | -0.194 | 0.045 | 0.044 | <0.001 | <0.001 |
| dmeadow | -0.257 | -0.103 | 0.013 | 0.016 | <0.001 | <0.001 |
| droad | 0.229 | 0.076 | 0.017 | 0.018 | <0.001 | <0.001 |
| dtrail | 4.210 | 0.939 | 0.226 | 0.074 | <0.001 | <0.001 |
| dwater | 0.306 | 0.429 | 0.012 | 0.016 | <0.001 | <0.001 |
| elevation | -0.518 | -0.563 | 0.021 | 0.028 | <0.001 | <0.001 |
| crowncover | -0.515 | -0.779 | 0.019 | 0.026 | <0.001 | <0.001 |
| snowdepth | -0.529 | -0.118 | 0.018 | 0.023 | <0.001 | <0.001 |
| vrm | 0.080 | 0.000 | 0.011 | 0.015 | <0.001 | 0.985 |

*Selection coefficient estimates for each landscape covariate in use-availability resource selection functions modelling seasonal habitat selection (May 15–July 15) in parturient and non-parturient bighorn sheep ewes. A separate RSF was fit for each reproductive status. A random intercept was included for each animal in the models.*
