# Supplementary material for: Changes in movement, habitat use, and response to human disturbance accompany parturition events in bighorn sheep (Ovis canadensis)
Source: Mov Ecol. 2023 Jul 4;11:36. doi: 10.1186/s40462-023-00404-2 (PMC10318713; doi:10.1186/s40462-023-00404-2)
Supplement: Supplementary file 2 — Additional file 2. HMM results for each individual sheep. This file contains the figures with the movement metrics, predicted latent states, and lambing datefor each individual sheep in the test and training datasets, organized by reproductive status. [file 40462_2023_404_MOESM2_ESM.pdf]

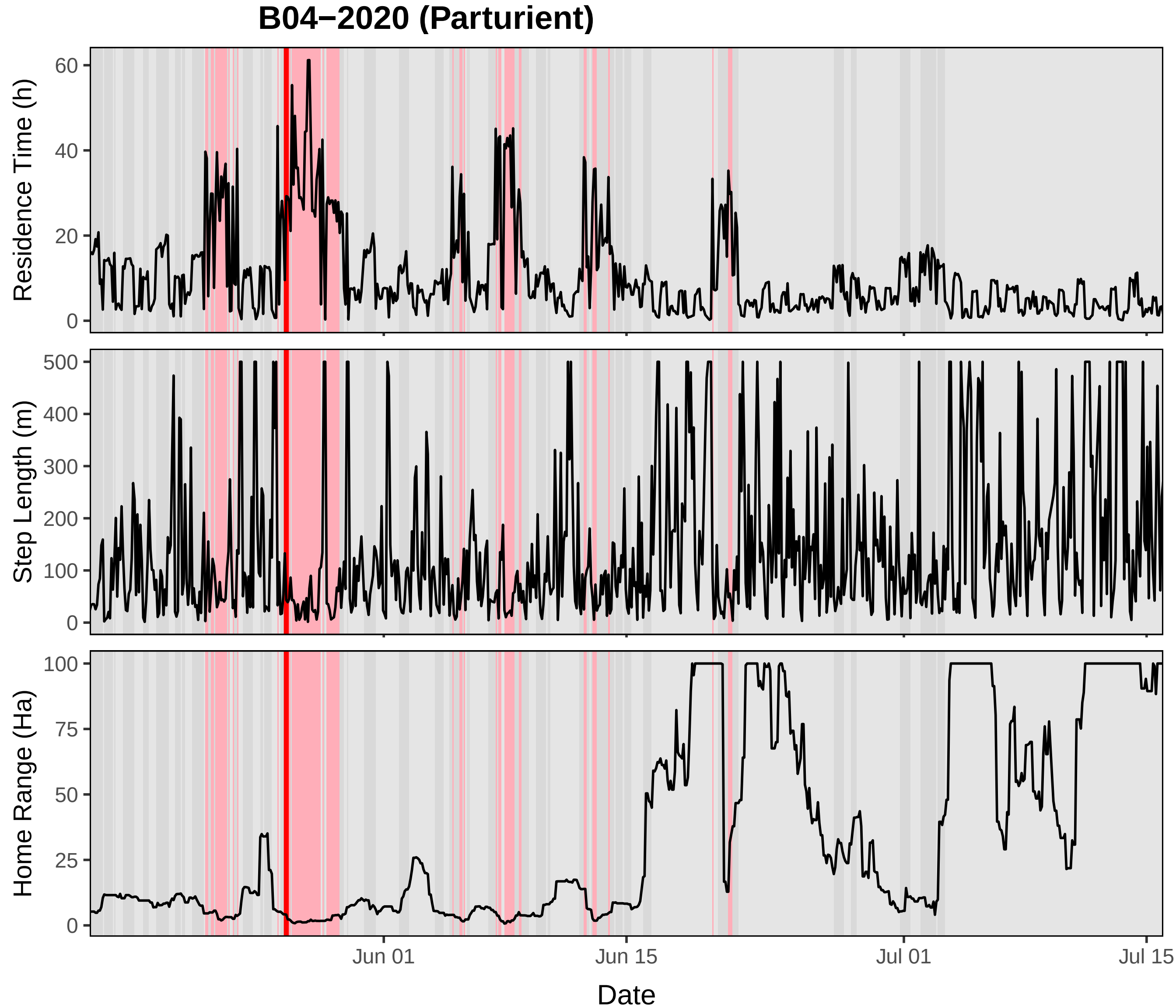
B04−2020 (Parturient)
Residence Time (h)
60
40
20
0
Step Length (m)
500
400
300
200
100
0
Home Range (Ha)
100
75
50
25
0
Jun 01
Jun 15
Jul 01
Jul 15
Date

B05−2022 (Parturient)

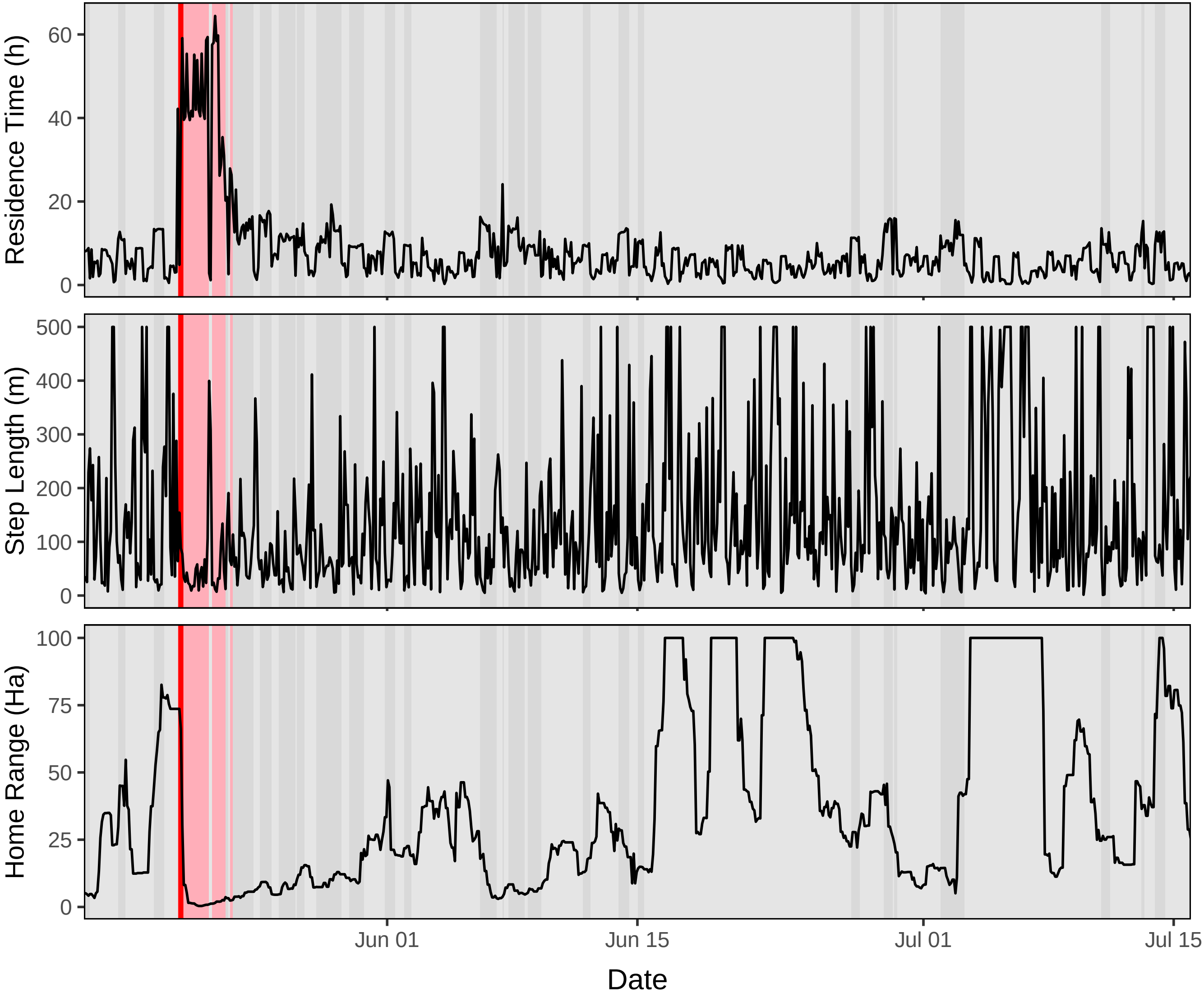

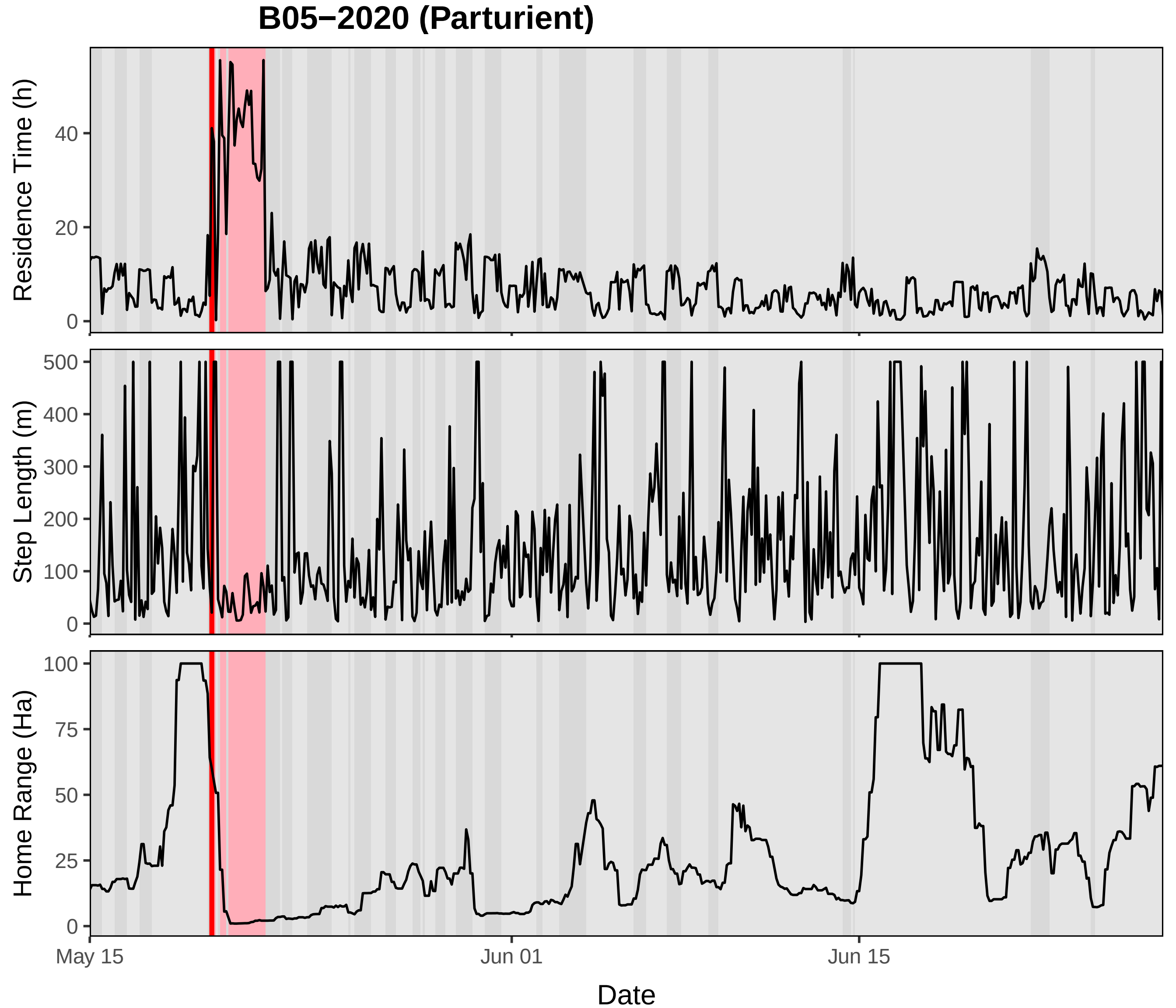
B05−2020 (Parturient)
Residence Time (h)
0
20
40
Step Length (m)
0
100
200
300
400
500
Home Range (Ha)
0
25
50
75
100
May 15
Jun 01
Jun 15
Date

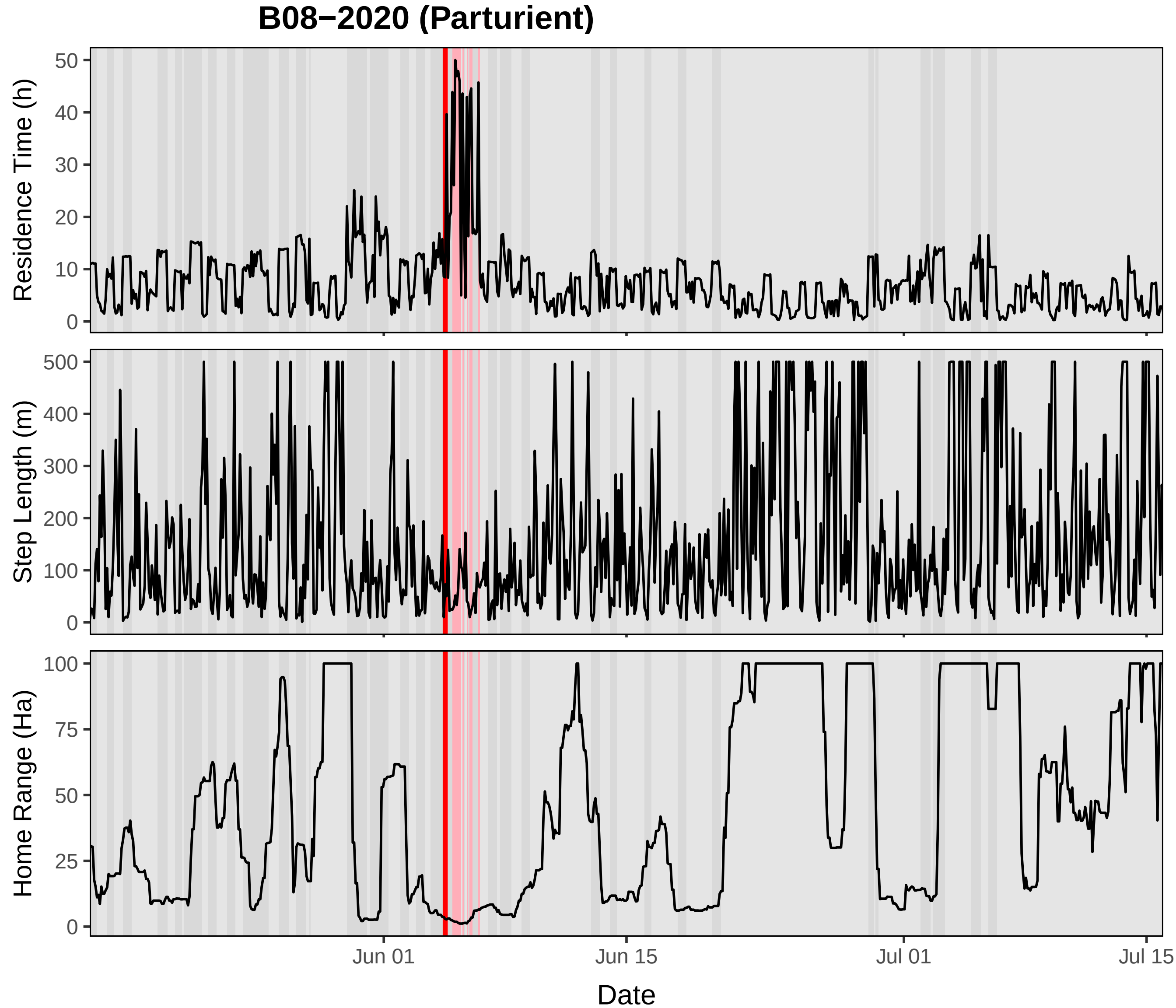
B08−2020 (Parturient)
Residence Time (h)
50
40
30
20
10
0
Step Length (m)
500
400
300
200
100
0
Home Range (Ha)
100
75
50
25
0
Jun 01
Jun 15
Jul 01
Jul 15
Date

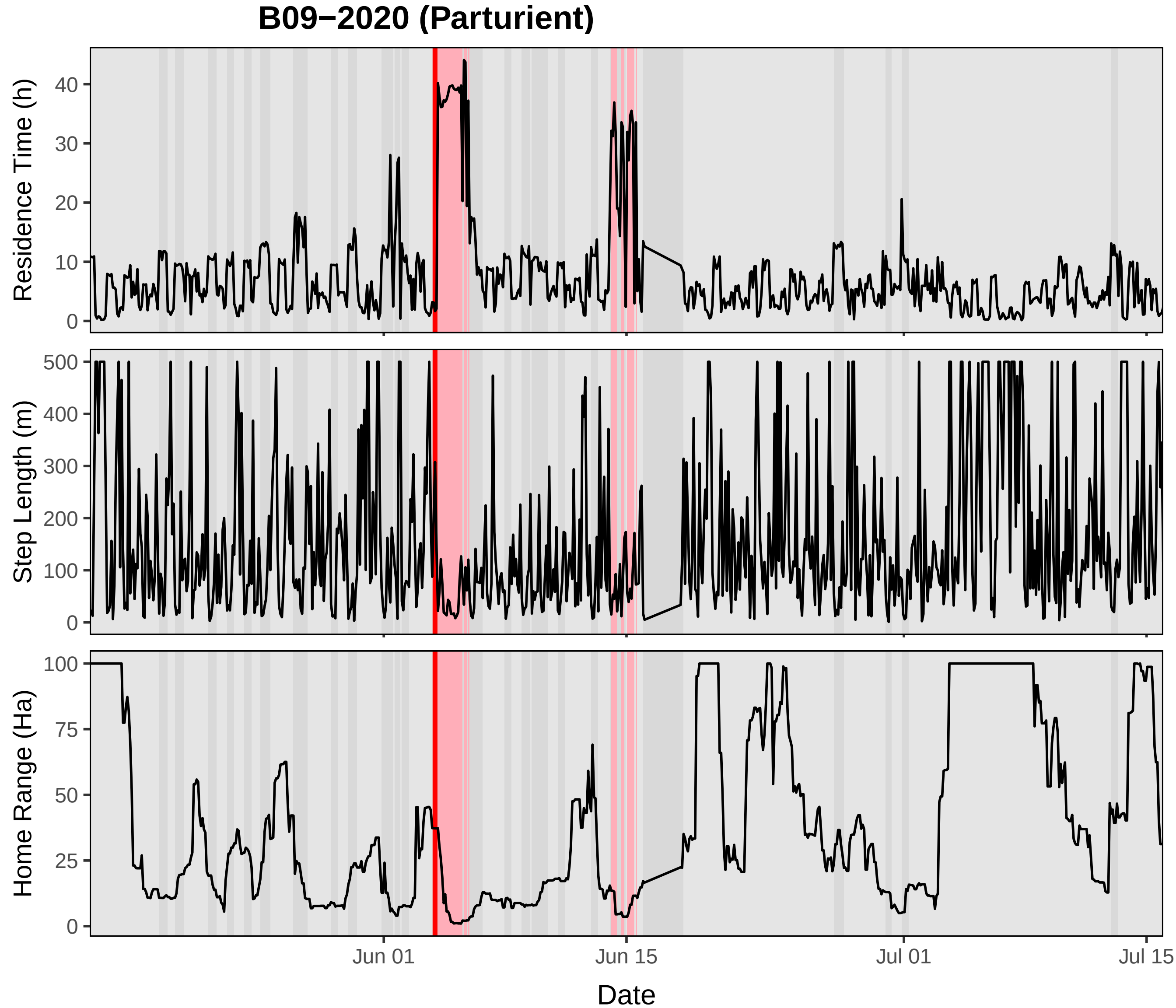
B09−2020 (Parturient)
Residence Time (h)
0
10
20
30
40
Step Length (m)
0
100
200
300
400
500
Home Range (Ha)
0
25
50
75
100
Jun 01
Jun 15
Jul 01
Jul 15
Date

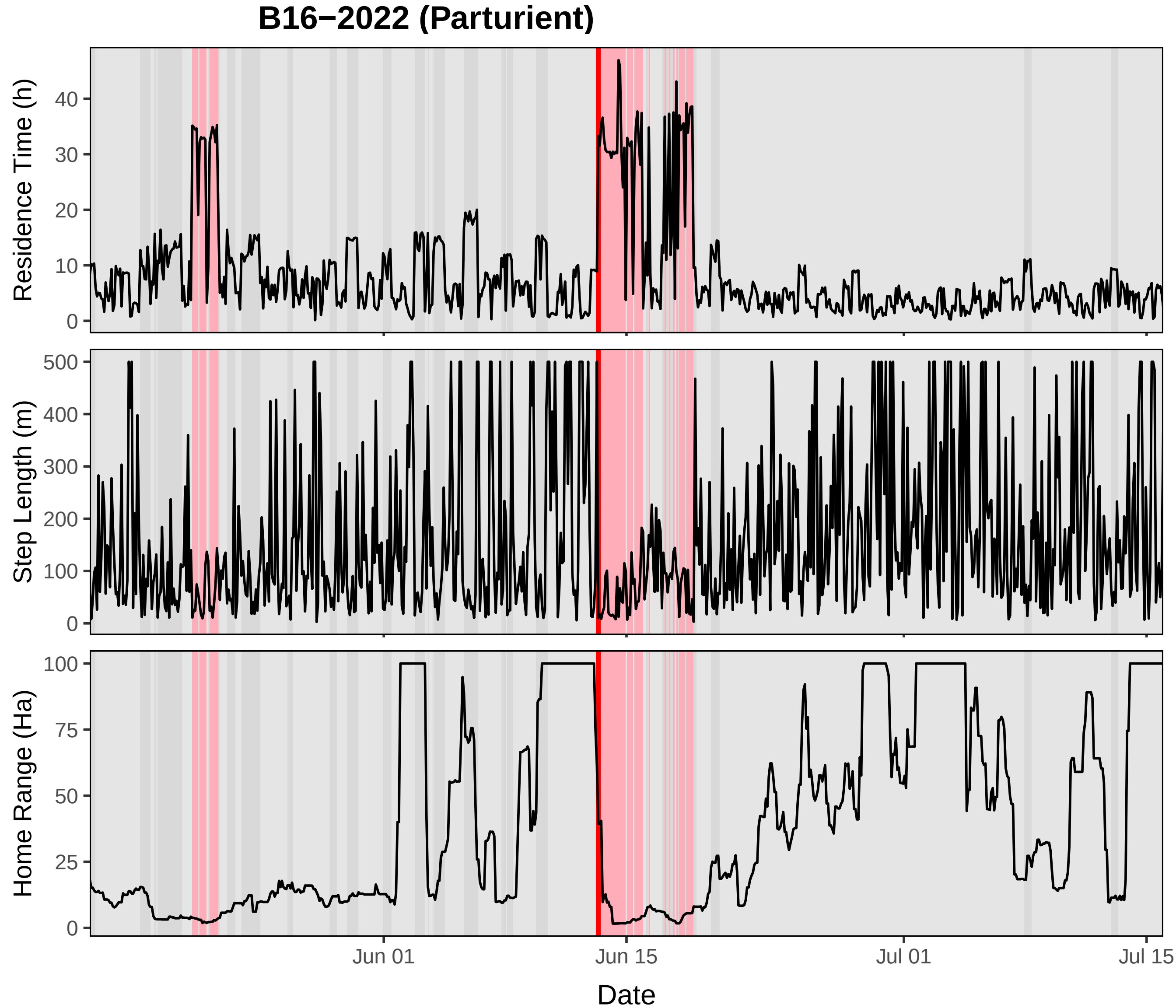

B16−2022 (Parturient)
Residence Time (h)
0
10
20
30
40
Step Length (m)
0
100
200
300
400
500
Home Range (Ha)
0
25
50
75
100
Jun 01
Jun 15
Jul 01
Jul 15
Date

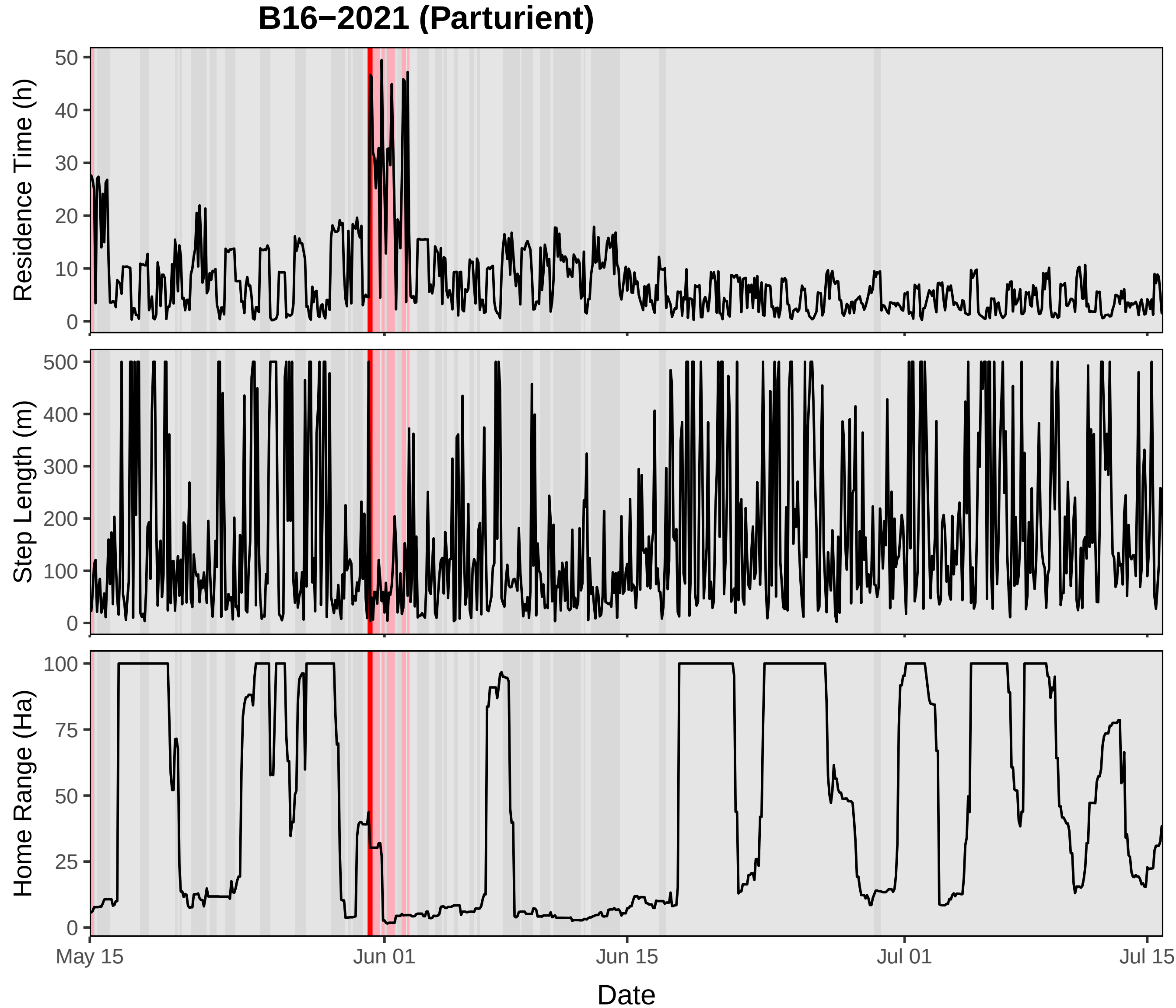

B16−2021 (Parturient)
Residence Time (h)
Step Length (m)
Home Range (Ha)
Date
May 15
Jun 01
Jun 15
Jul 01
Jul 15

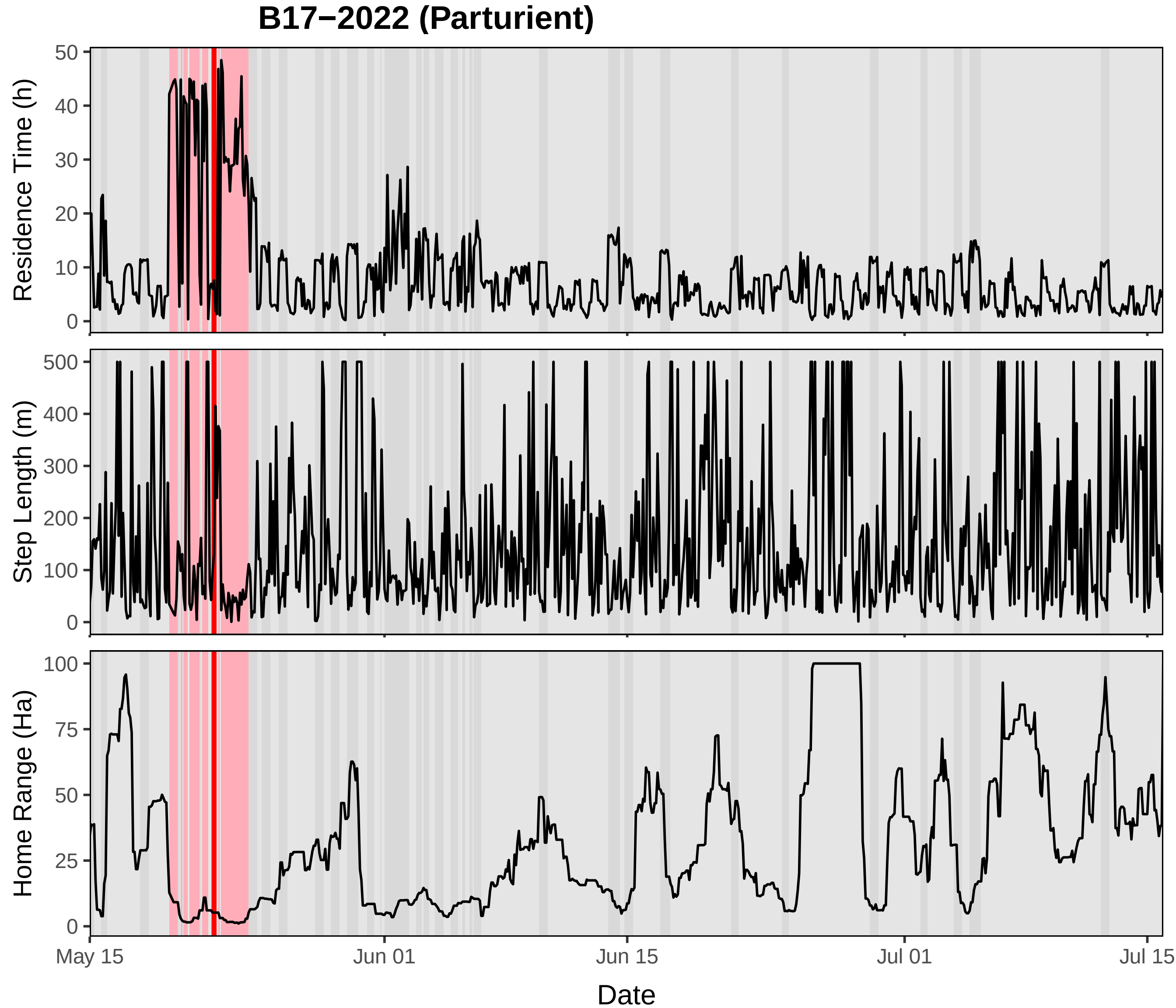

B17−2022 (Parturient)
Residence Time (h)
Step Length (m)
Home Range (Ha)
Date
May 15
Jun 01
Jun 15
Jul 01
Jul 15

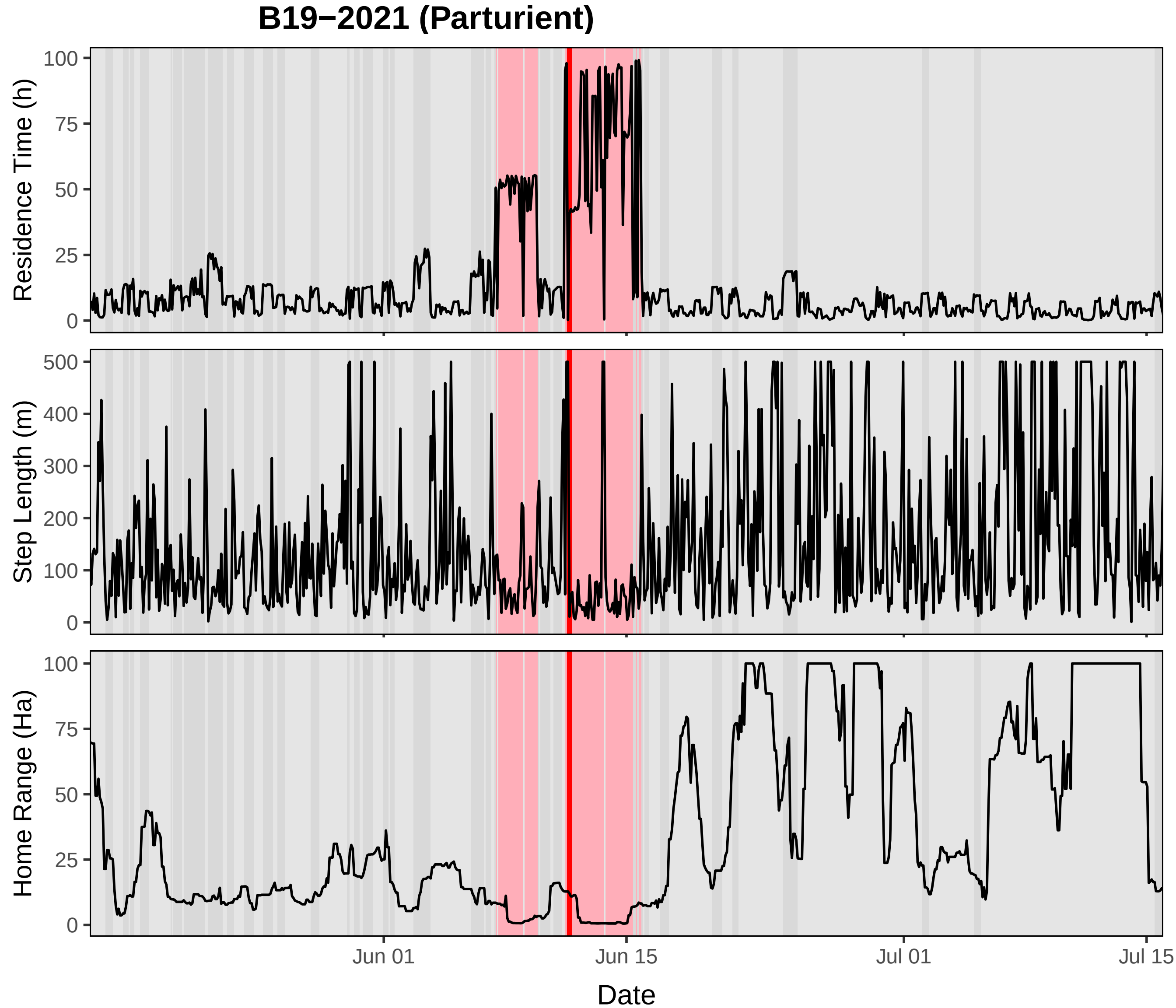
B19−2021 (Parturient)
Residence Time (h)
Step Length (m)
Home Range (Ha)
Date
Jun 01
Jun 15
Jul 01
Jul 15
0
25
50
75
100
0
100
200
300
400
500

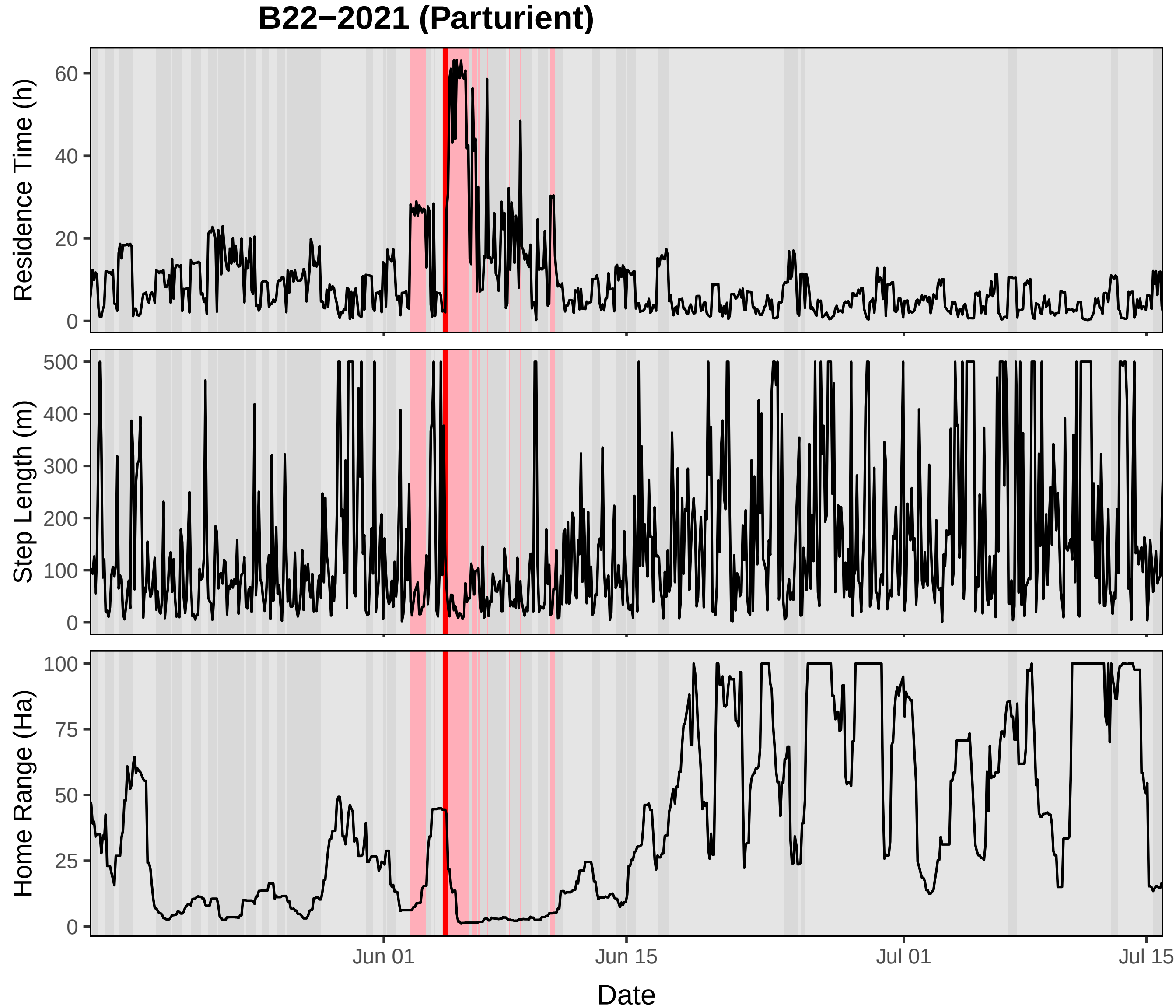
B22−2021 (Parturient)
Residence Time (h)
Step Length (m)
Home Range (Ha)
Date
Jun 01
Jun 15
Jul 01
Jul 15

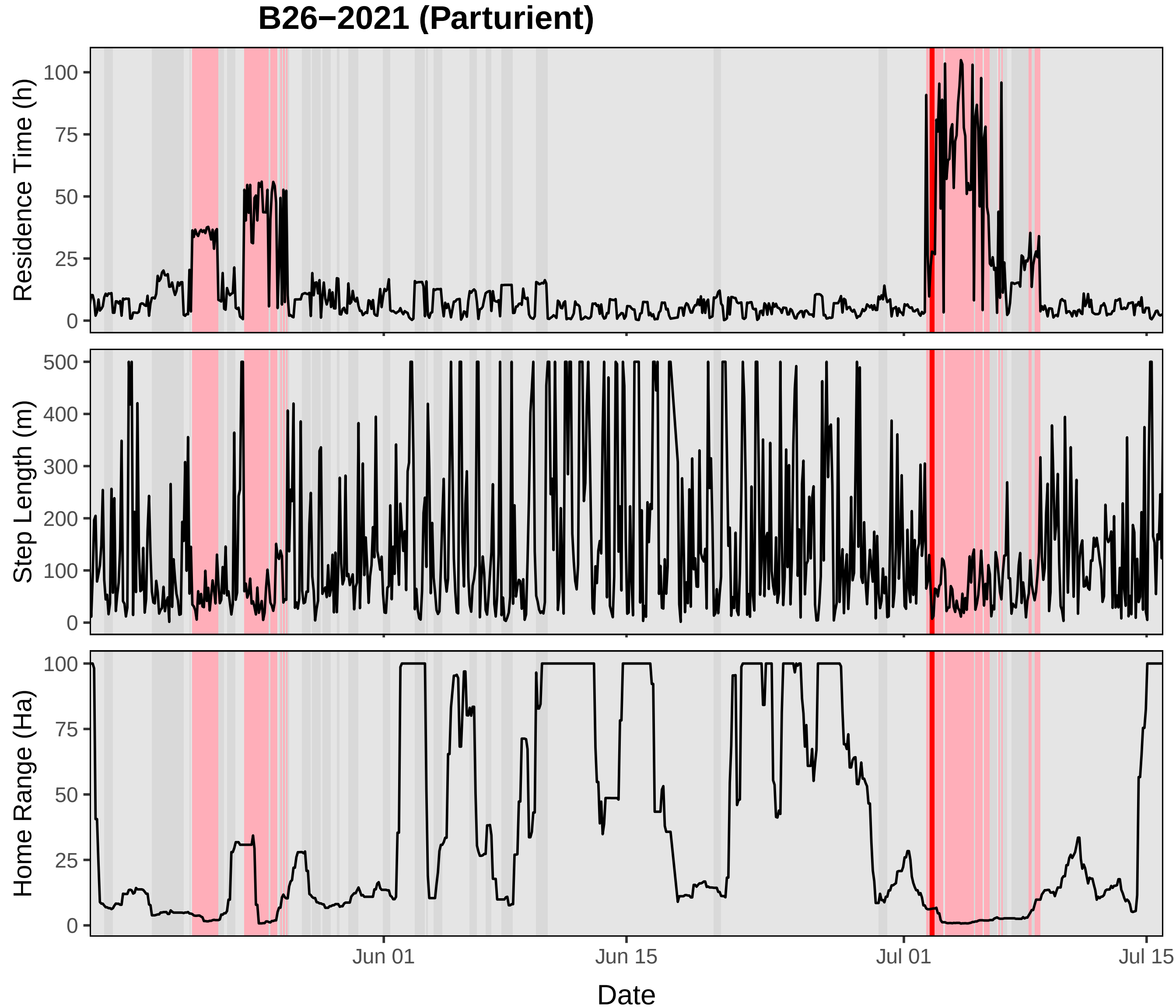

B26−2021 (Parturient)
Residence Time (h)
100
75
50
25
0
Step Length (m)
500
400
300
200
100
0
Home Range (Ha)
100
75
50
25
0
Jun 01
Jun 15
Jul 01
Jul 15
Date

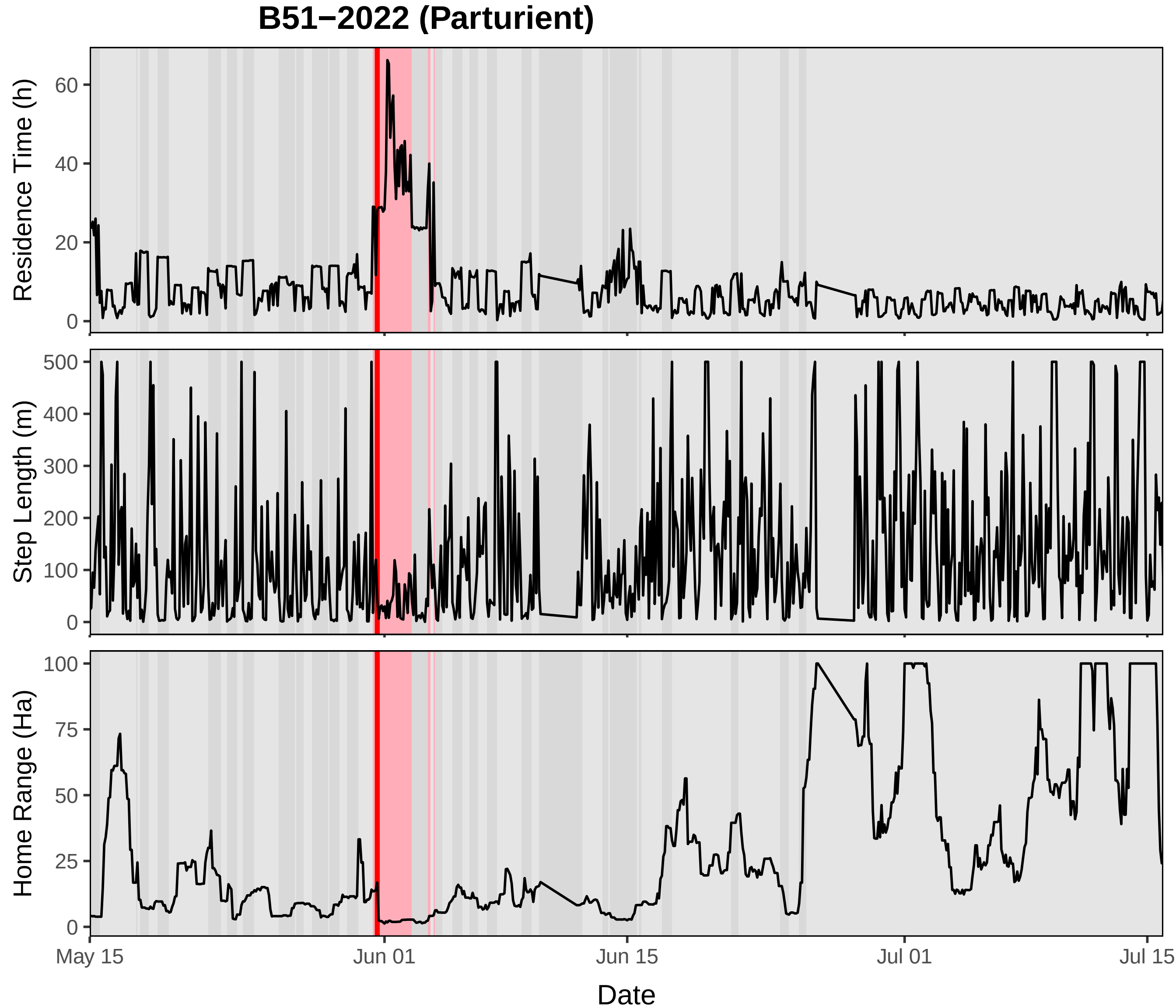

B51−2022 (Parturient)
Residence Time (h)
0
20
40
60
Step Length (m)
0
100
200
300
400
500
Home Range (Ha)
0
25
50
75
100
May 15
Jun 01
Jun 15
Jul 01
Jul 15
Date

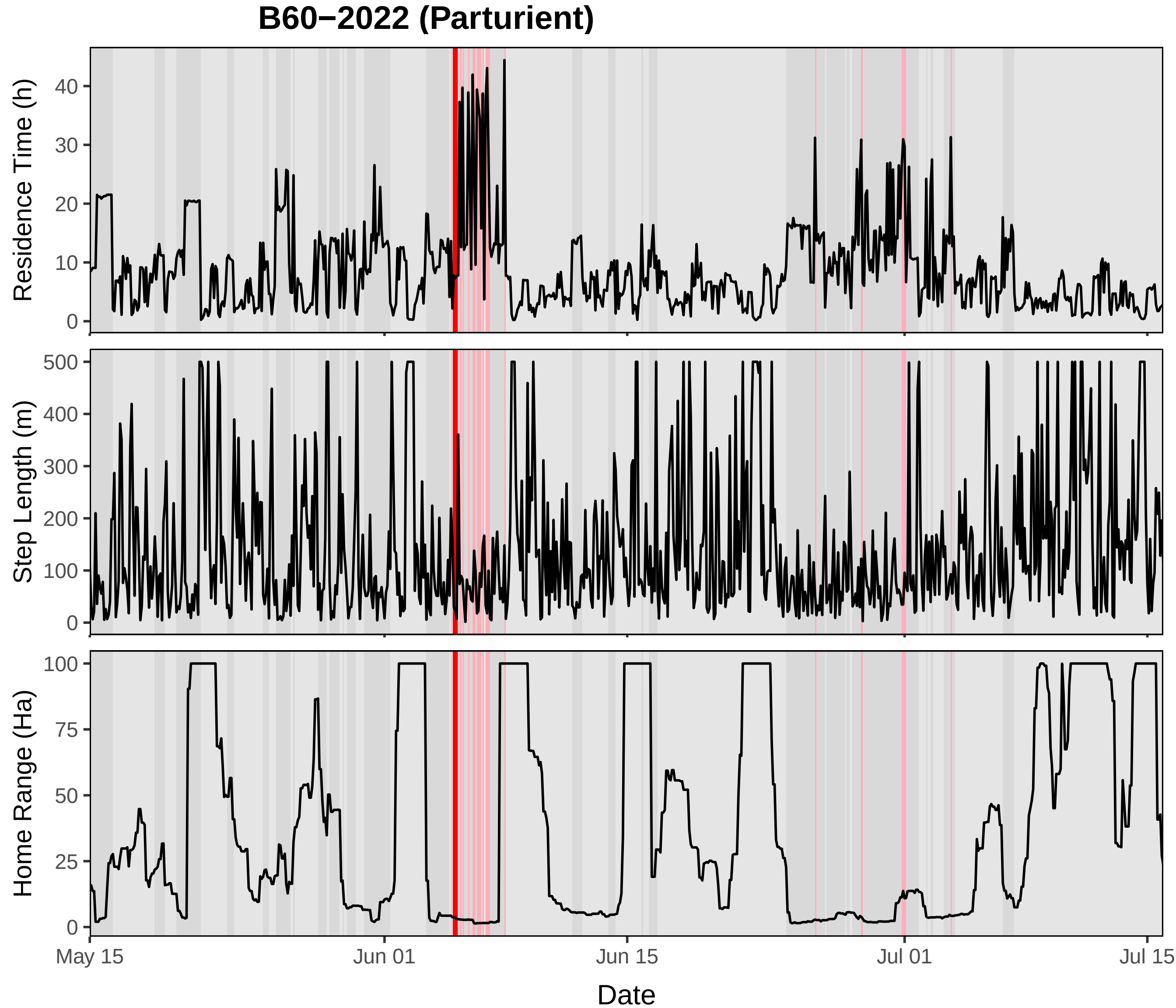
B60−2022 (Parturient)
Residence Time (h)
Step Length (m)
Home Range (Ha)
Date
May 15
Jun 01
Jun 15
Jul 01
Jul 15

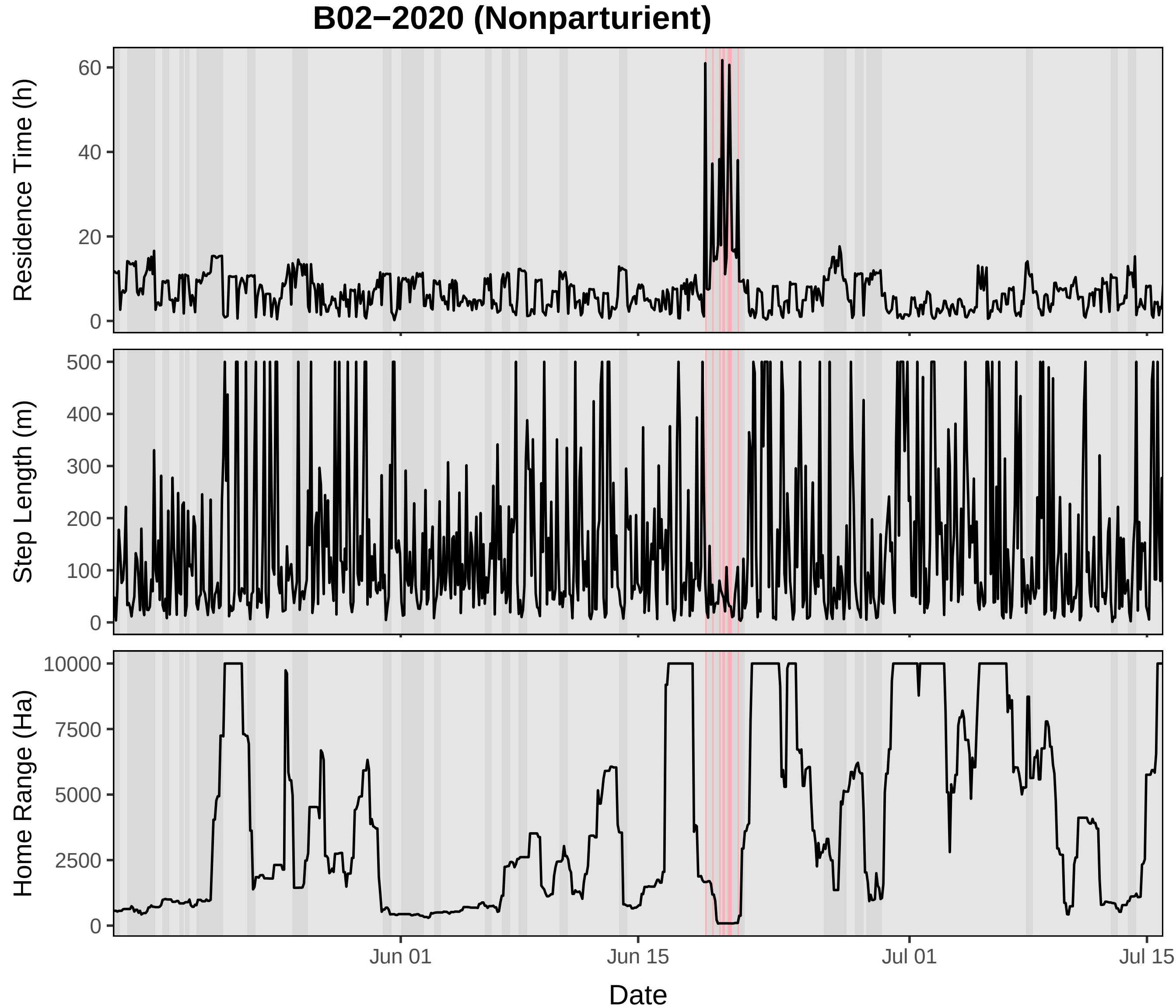

B02−2020 (Nonparturient)
Residence Time (h)
60
40
20
0
Step Length (m)
500
400
300
200
100
0
Home Range (Ha)
10000
7500
5000
2500
0
Jun 01
Jun 15
Jul 01
Jul 15
Date

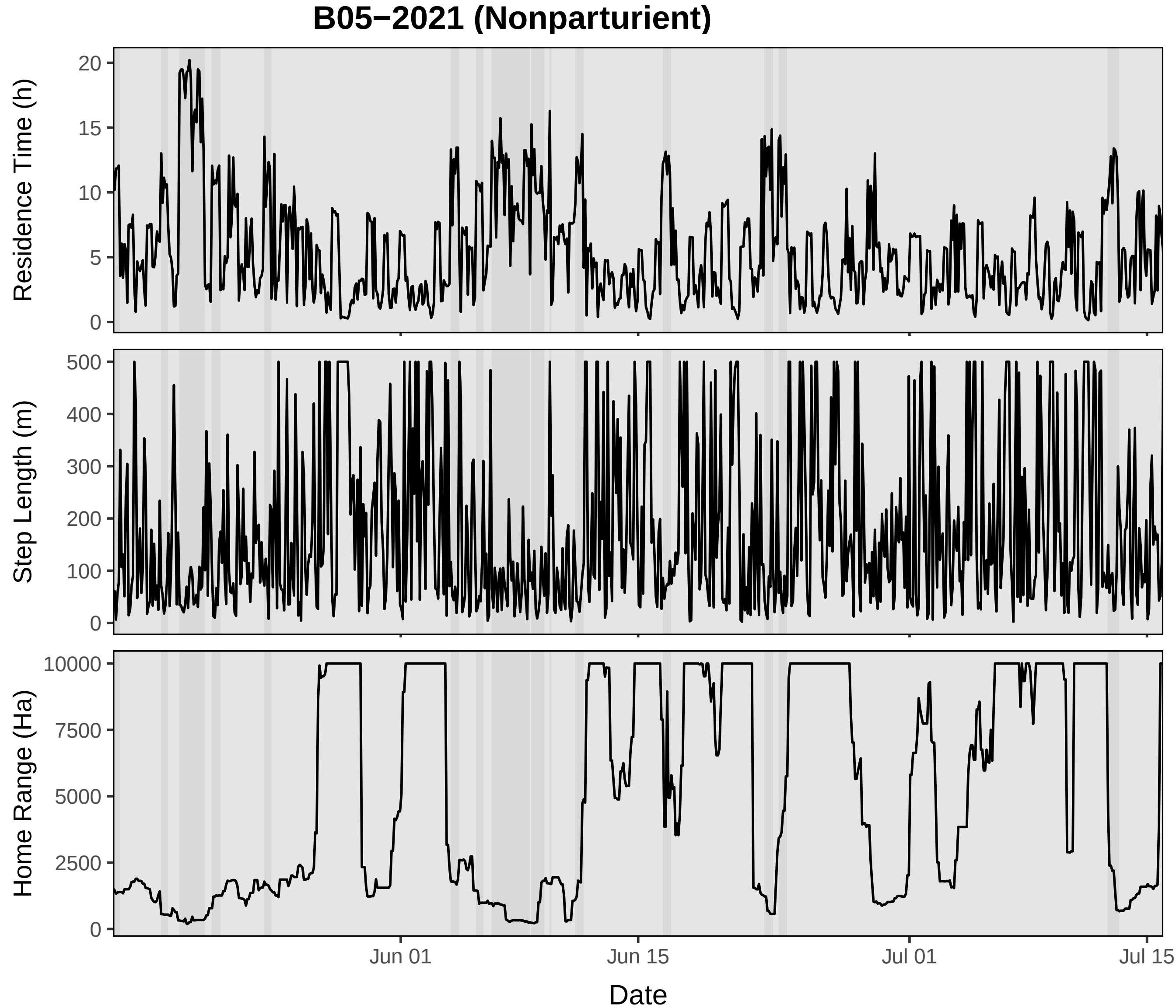

B05−2021 (Nonparturient)
Residence Time (h)
20
15
10
5
0
Step Length (m)
500
400
300
200
100
0
Home Range (Ha)
10000
7500
5000
2500
0
Jun 01
Jun 15
Jul 01
Jul 15
Date

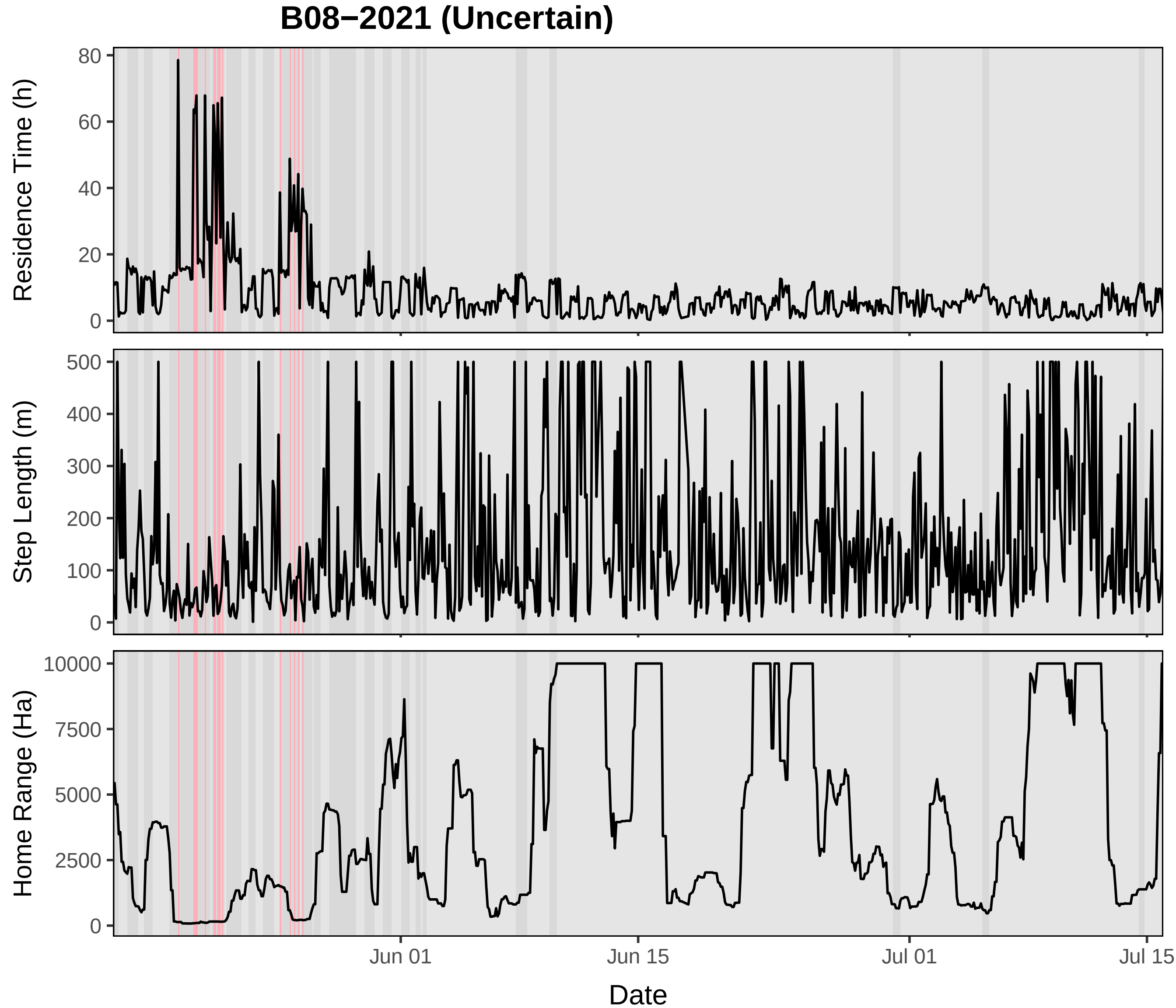
B08−2021 (Uncertain)
Residence Time (h)
0
20
40
60
80
Step Length (m)
0
100
200
300
400
500
Home Range (Ha)
0
2500
5000
7500
10000
Jun 01
Jun 15
Jul 01
Jul 15
Date

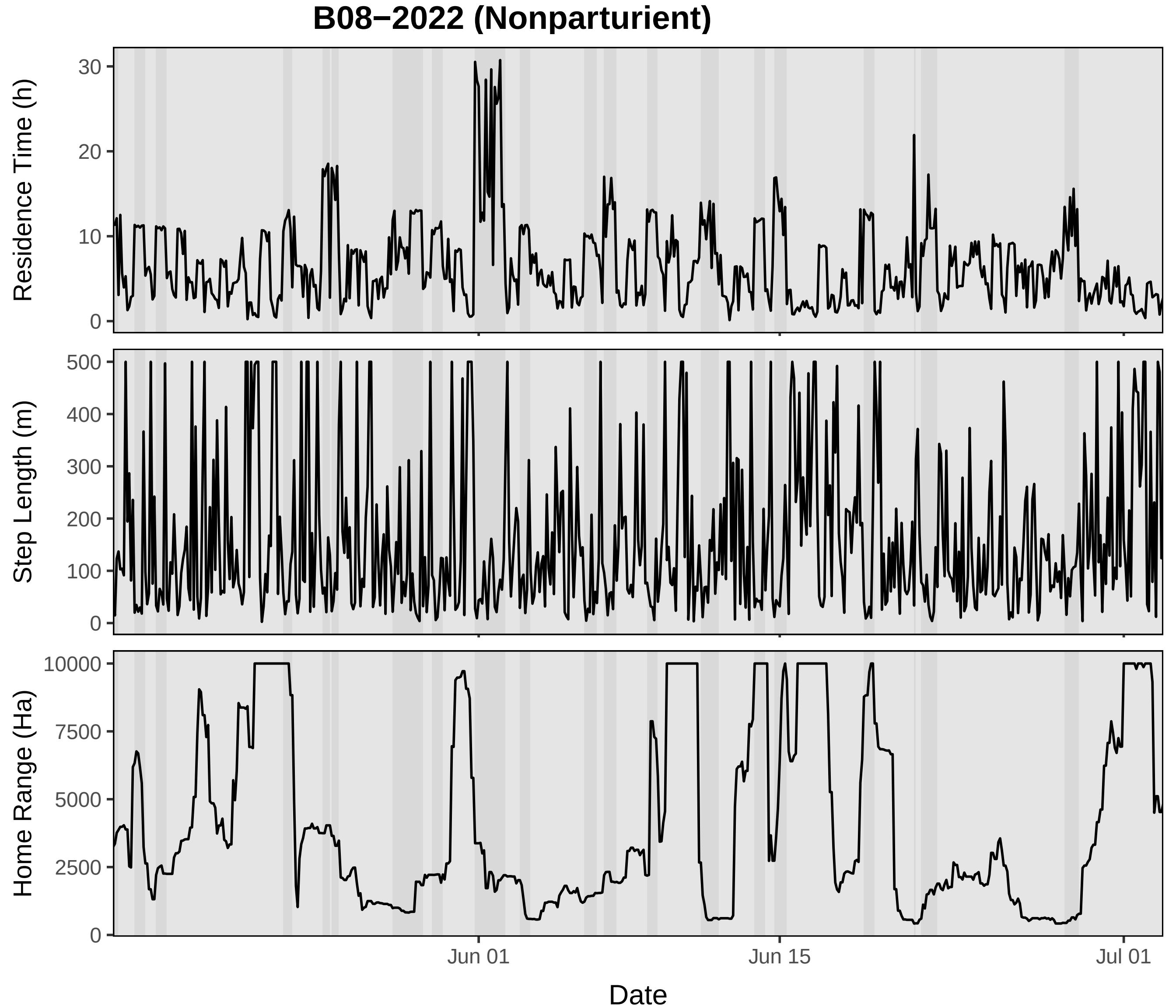
B08−2022 (Nonparturient)
Residence Time (h)
30
20
10
0
Step Length (m)
500
400
300
200
100
0
Home Range (Ha)
10000
7500
5000
2500
0
Jun 01
Jun 15
Jul 01
Date

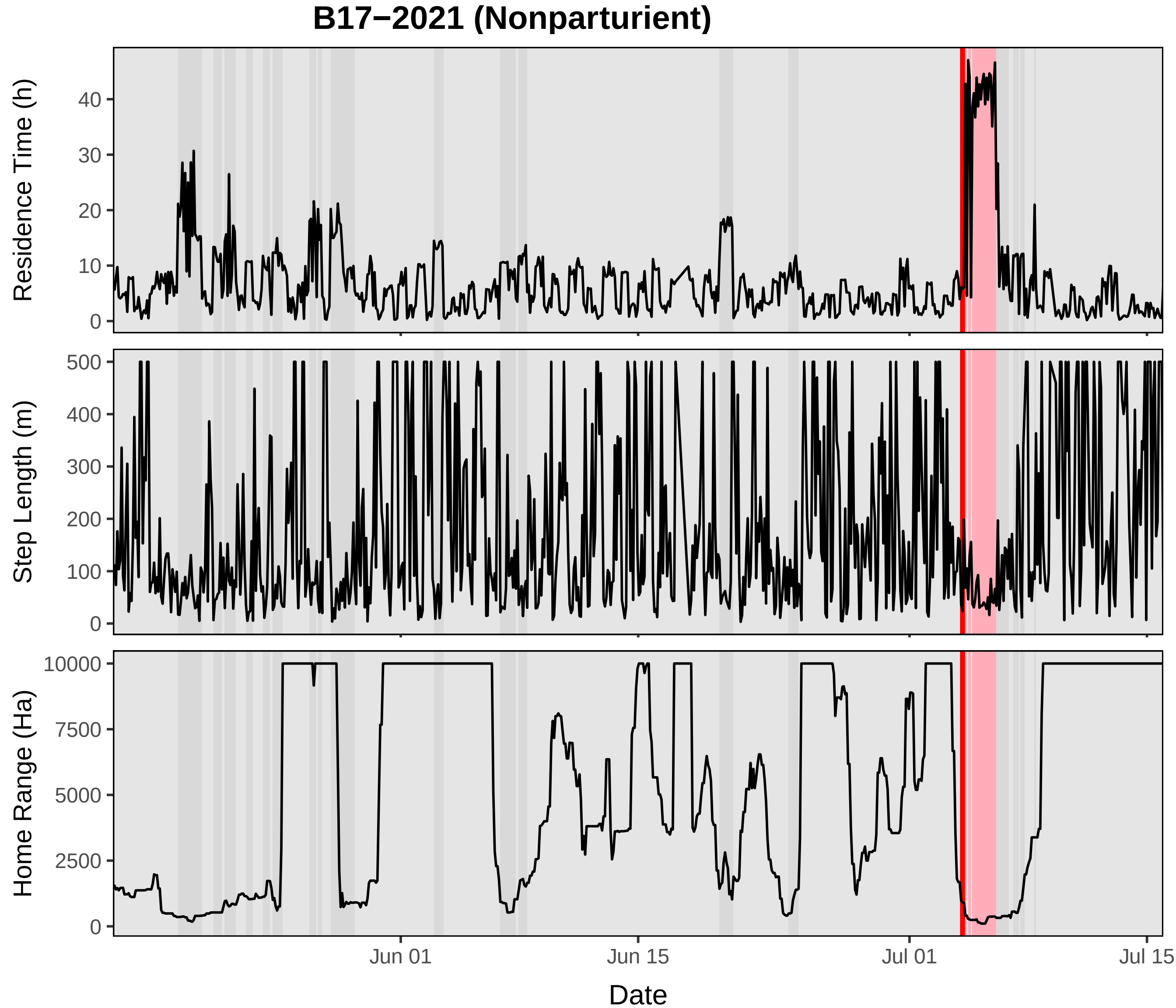
B17−2021 (Nonparturient)
Residence Time (h)
0
10
20
30
40
Step Length (m)
0
100
200
300
400
500
Home Range (Ha)
0
2500
5000
7500
10000
Jun 01
Jun 15
Jul 01
Jul 15
Date

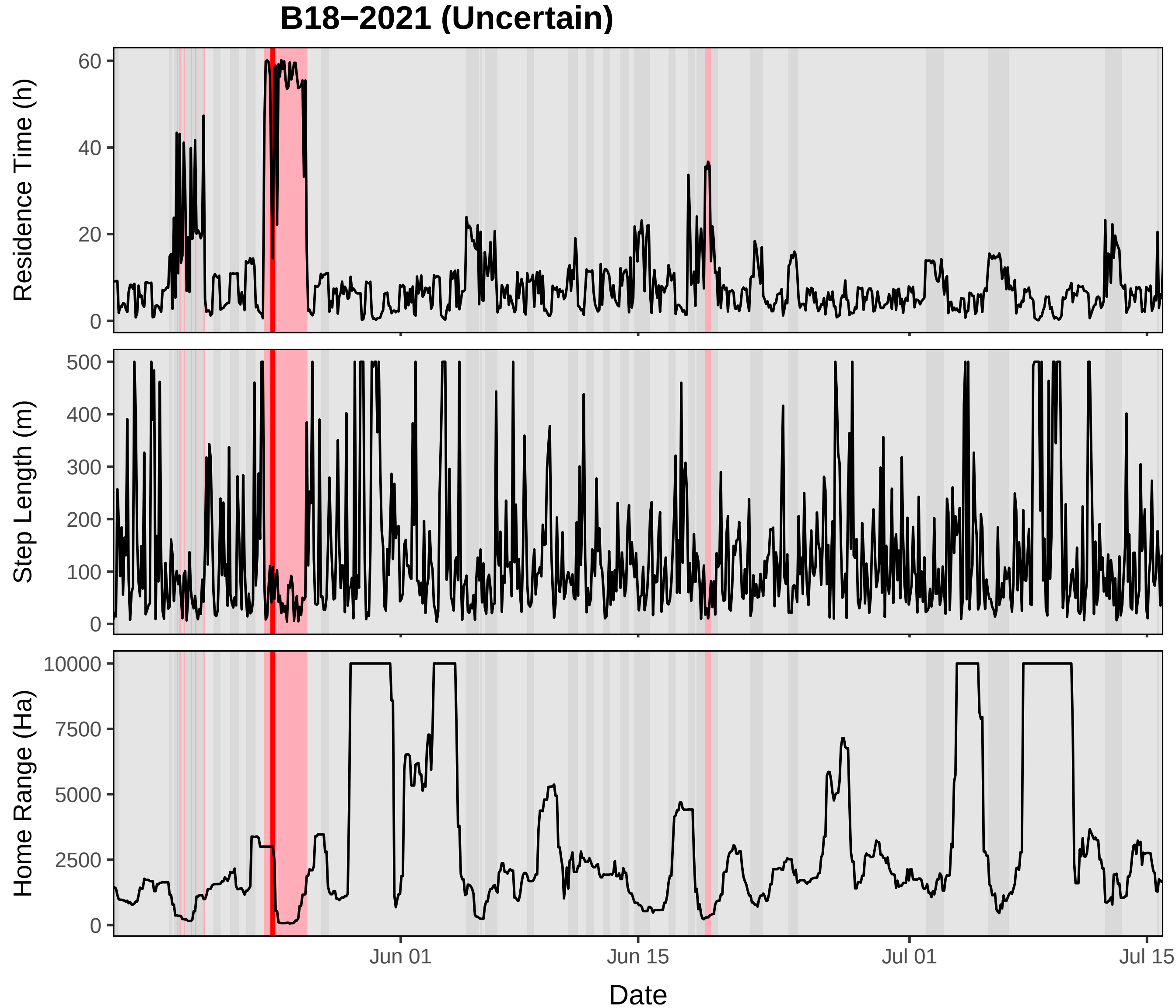
B18−2021 (Uncertain)
Residence Time (h)
0
20
40
60
Step Length (m)
0
100
200
300
400
500
Home Range (Ha)
0
2500
5000
7500
10000
Jun 01
Jun 15
Jul 01
Jul 15
Date

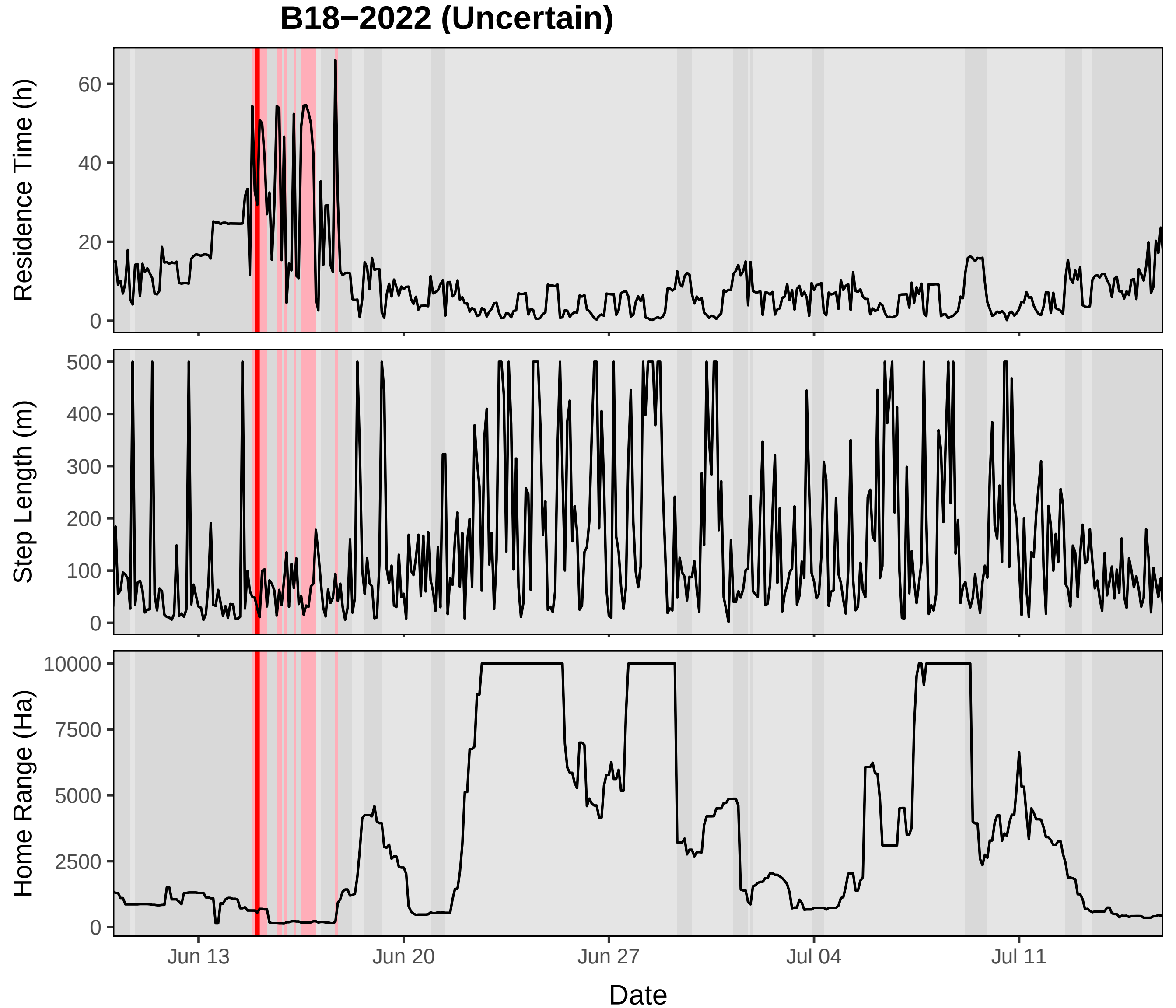

B18−2022 (Uncertain)
Residence Time (h)
Step Length (m)
Home Range (Ha)
Date
Jun 13
Jun 20
Jun 27
Jul 04
Jul 11

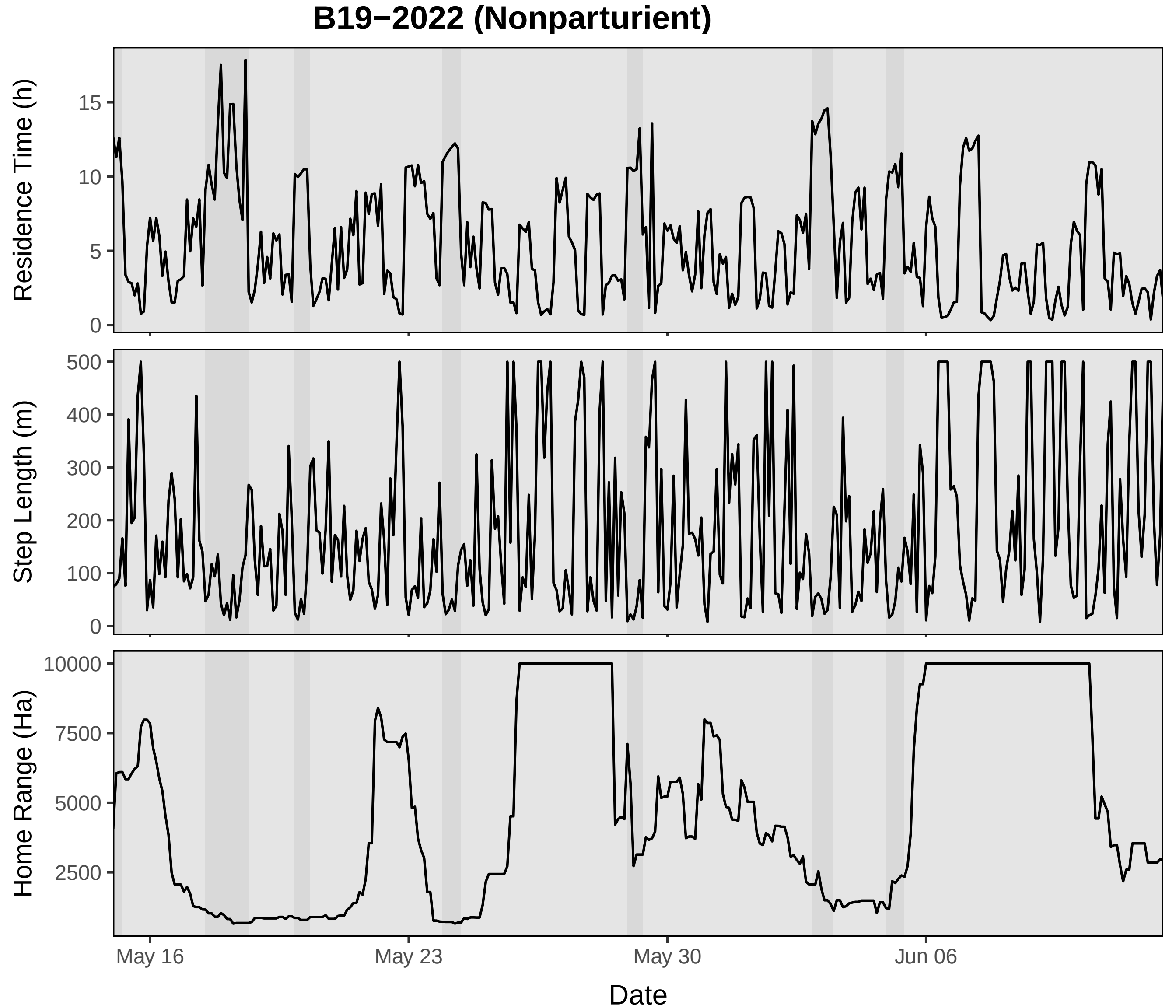
B19−2022 (Nonparturient)
Residence Time (h)
0
5
10
15
Step Length (m)
0
100
200
300
400
500
Home Range (Ha)
2500
5000
7500
10000
May 16
May 23
May 30
Jun 06
Date

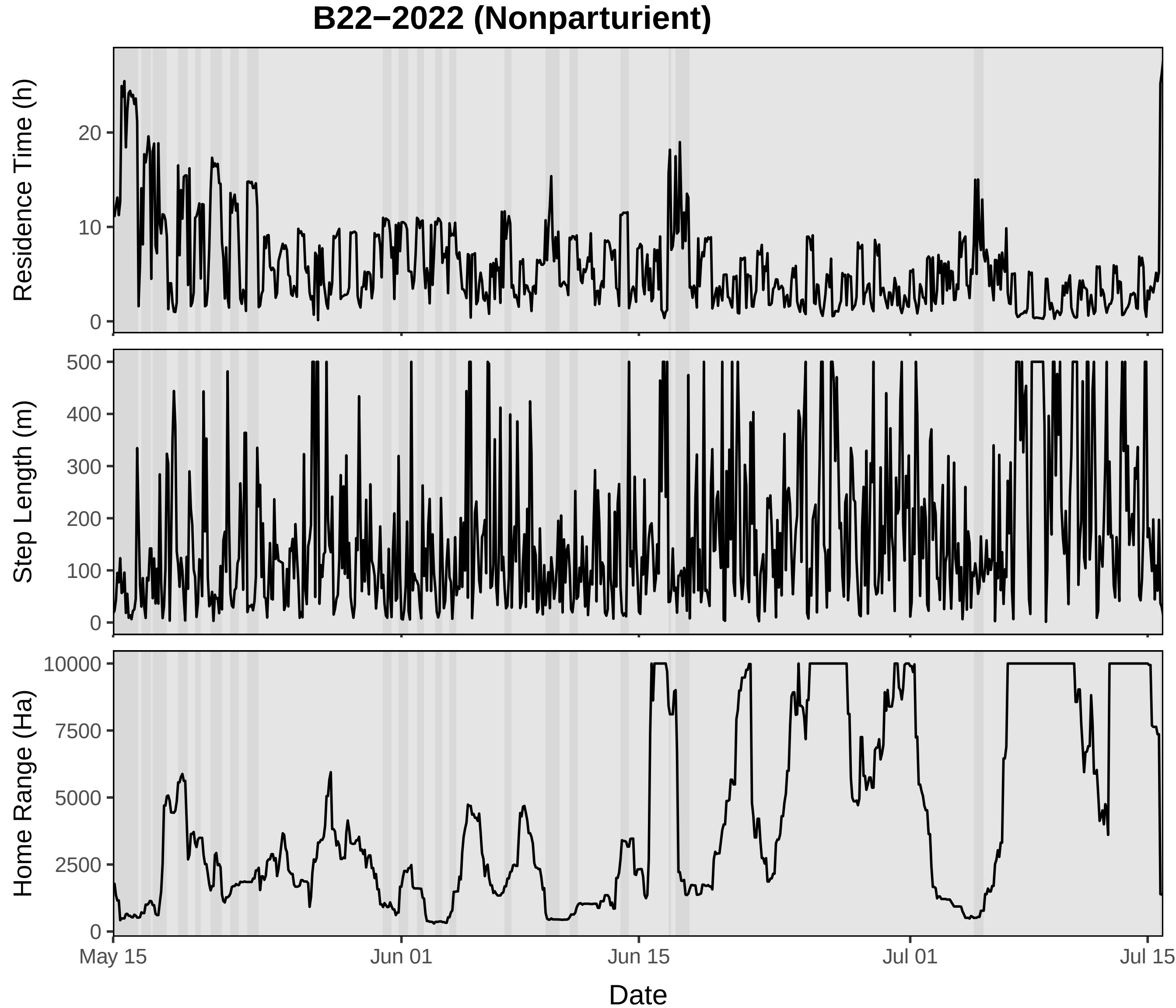
B22−2022 (Nonparturient)
Residence Time (h)
0
10
20
Step Length (m)
0
100
200
300
400
500
Home Range (Ha)
0
2500
5000
7500
10000
May 15
Jun 01
Jun 15
Jul 01
Jul 15
Date

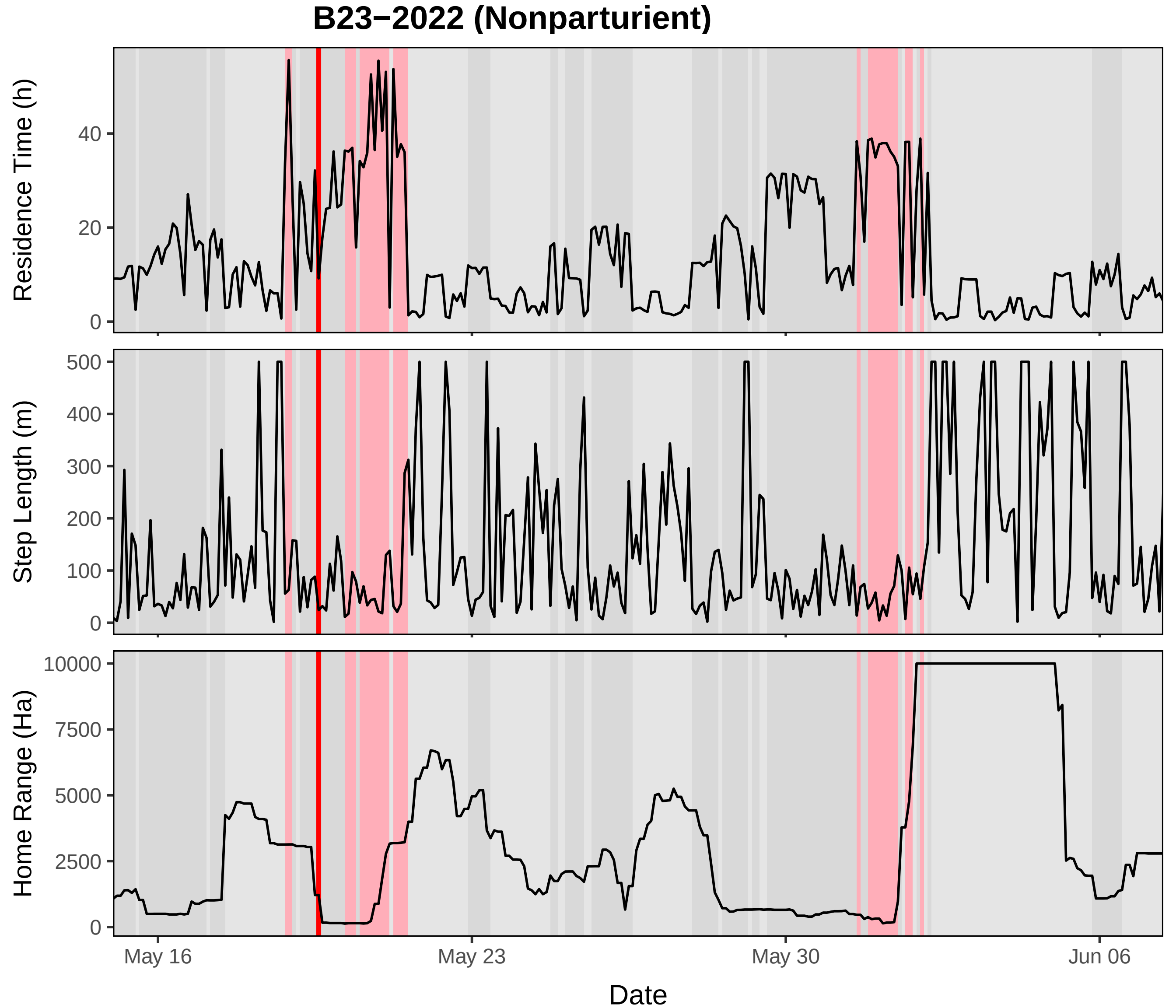

B23−2022 (Nonparturient)
Residence Time (h)
0
20
40
Step Length (m)
0
100
200
300
400
500
Home Range (Ha)
0
2500
5000
7500
10000
May 16
May 23
May 30
Jun 06
Date

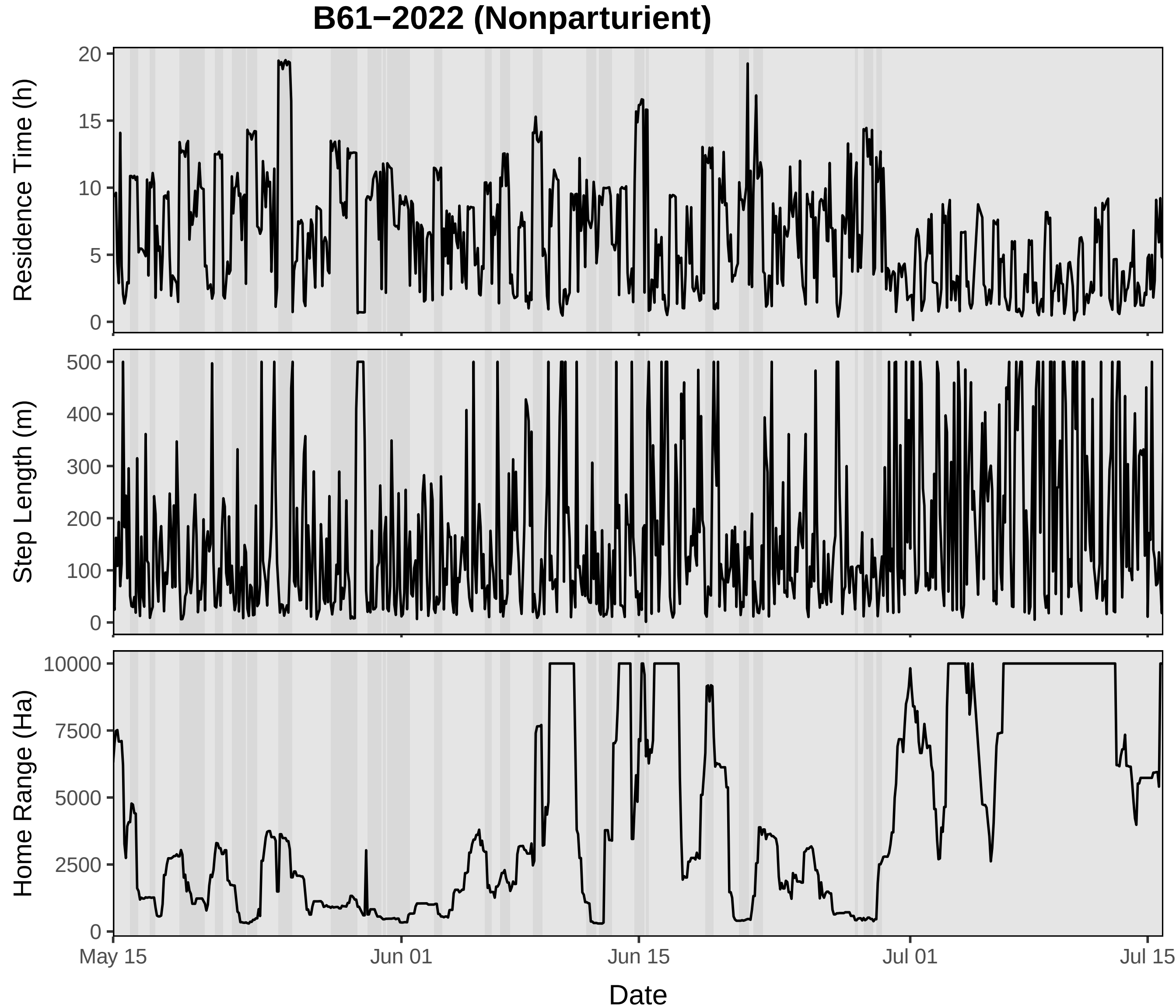
B61−2022 (Nonparturient)
Residence Time (h)
20
15
10
5
0
Step Length (m)
500
400
300
200
100
0
Home Range (Ha)
10000
7500
5000
2500
0
May 15
Jun 01
Jun 15
Jul 01
Jul 15
Date
